# Molecular Signature of Extracellular Vesicular Small Non-Coding RNAs Derived from Cerebrospinal Fluid of Leptomeningeal Metastasis Patients: Functional Implication of miR-21 and Other Small RNAs in Cancer Malignancy

**DOI:** 10.3390/cancers13020209

**Published:** 2021-01-08

**Authors:** Kyue-Yim Lee, Yoona Seo, Ji Hye Im, Jiho Rhim, Woosun Baek, Sewon Kim, Ji-Woong Kwon, Byong Chul Yoo, Sang Hoon Shin, Heon Yoo, Jong Bae Park, Ho-Shin Gwak, Jong Heon Kim

**Affiliations:** 1 Department of Cancer Control, National Cancer Center Graduate School of Cancer Science and Policy, Goyang 10408, Korea; 70564@ncc.re.kr (K.-Y.L.); 75262@ncc-gcsp.ac.kr (J.H.I.); 2 Neuro-Oncology Clinic, National Cancer Center, Goyang 10408, Korea; jwkwon@ncc.re.kr (J.-W.K.); nsshin@ncc.re.kr (S.H.S.); heonyoo@ncc.re.kr (H.Y.); 3 Department of Cancer Biomedical Science, National Cancer Center Graduate School of Cancer Science and Policy, Goyang 10408, Korea; yoona.seo@ncc.re.kr (Y.S.); jhrhim@ncc.re.kr (J.R.); bws@ncc.re.kr (W.B.); yoo_akh@ncc.re.kr (B.C.Y.); jbp@ncc.re.kr (J.B.P.); 4 Division of Cancer Biology, Research Institute, National Cancer Center, Goyang 10408, Korea; 5 Macrogen Inc., Seoul 08511, Korea; raphaelfriend30@gmail.com

**Keywords:** leptomeningeal metastasis, cerebrospinal fluid, extracellular vesicle, biomarker, RNA sequencing, small non-coding RNA, microRNA

## Abstract

**Simple Summary:**

Leptomeningeal metastasis (LM) is a lethal complication in which cancer metastasizes to the meninges. Currently, there are neither definitive treatments nor diagnosis methods for LM patients. In this study, we suggest the examination of small non-coding RNA (smRNA) populations of extracellular vesicles (EVs) derived from the cerebrospinal fluid (CSF) as a potential vehicle for diagnosis and treatment strategies. Systemic and quantitative analysis of smRNA subpopulations from LM CSF EVs showed unique expression patterns between LM patients and healthy donors. In addition, LM CSF EVs smRNAs appeared to be associated with LM pathogenesis suggesting they may be viable targets for novel diagnostic and treatment strategies.

**Abstract:**

Leptomeningeal metastasis (LM) is a fatal and rare complication of cancer in which the cancer spreads via the cerebrospinal fluid (CSF). At present, there is no definitive treatment or diagnosis for this deleterious disease. In this study, we systemically and quantitatively investigated biased expression of key small non-coding RNA (smRNA) subpopulations from LM CSF extracellular vesicles (EVs) via a unique smRNA sequencing method. The analyzed subpopulations included microRNA (miRNA), Piwi-interacting RNA (piRNA), Y RNA, small nuclear RNA (snRNA), small nucleolar RNAs (snoRNA), vault RNA (vtRNA), novel miRNA, etc. Here, among identified miRNAs, miR-21, which was already known to play an essential oncogenic role in tumorigenesis, was thoroughly investigated via systemic biochemical, miR-21 sensor, and physiological cell-based approaches, with the goal of confirming its functionality and potential as a biomarker for the pathogenesis and diagnosis of LM. We herein uncovered LM CSF extravesicular smRNAs that may be associated with LM-related complications and elucidated plausible pathways that may mechanistically contribute to LM progression. In sum, the analyzed smRNA subpopulations will be useful as targets for the development of therapeutic and diagnostic strategies for LM and LM-related complications.

## 1. Introduction

Leptomeningeal metastasis (LM) is a fatal and rare complication of cancer in which the cancer spreads to the meninges surrounding the brain and spinal cord via the cerebrospinal fluid (CSF) [1,2]. LM occurs in approximately 5% of people with cancer and is usually a terminal-stage cancer [1,2,3]. The median survival of LM patients is approximately 4–8 weeks [4]. At present, there is no definitive treatment or diagnosis of this deleterious disease. CSF is a clear, colorless body fluid found in the brain and spinal cord. It is produced by specialized ependymal cells in the choroid plexuses of the ventricles of the brain [1]. CSF acts as a cushion or buffer, providing basic mechanical and immunological protection to the brain inside the skull [5]. As a potential vehicle for the spread of LM, CSF can be considered a good resource for the identification of new biological markers for the diagnosis derived from LM cells [6,7,8].

Extracellular vesicles (EVs) mostly consist of exosomes and microvesicles (MVs) of different origin; however, there are limitations in current techniques to completely separate the MV from the exosome since they are overlapped in size and share surface biomarkers [9,10]. EVs are thought to function in intercellular communication. They contain not only essential macromolecules (e.g., proteins and lipids) from their cell of origin, but also functional RNA molecules that can be delivered to recipient cells to undergo translation or perform other functions [11].

Most of the RNAs found in EVs are small non-coding RNAs (smRNAs) of less than 200-nucleotides (nt) in length [12]. Recent studies have indicated that the smRNAs contained within EVs are generally enriched for functional species, such as the well-studied microRNAs (miRNAs) [13]. This finding suggests that EVs are likely to have a direct influence on gene expression of recipient cells upon internalization [14]. In addition to miRNAs, the advent of next-generation sequencing (NGS) revealed the presence of a broad spectrum of additional smRNAs in cells, most of which may be incorporated into EVs [13]. These additional smRNAs include Piwi-interacting RNA (piRNA), Y RNA, small nuclear RNA (snRNA), small nucleolar RNA (snoRNA), vault RNA (vtRNA), tRNA-derived small RNA (tsRNA), ribosomal RNA (rRNA), and small interfering RNA (siRNA) [15]. So far, only the miRNAs have been confirmed to sustain gene-regulatory functions upon cell-to-cell transfer [16]. Thus, researchers have focused on EV-related miRNAs for potential therapeutic exploitation.

miRNAs are considered to be strong prognostic markers and key therapeutic targets in various human diseases, especially cancer [17,18]. More than 600 different miRNAs encoded in the human genome negatively regulate gene expression at the posttranscriptional level by inducing translational repression and/or destabilizing specific mRNAs by targeting their 3′ untranslated regions (UTRs) [19]. Thousands of different genes can be subject to regulation by a single miRNA or miRNA family [20]. Although the action mechanisms and oncogenic roles of miRNAs are relatively well understood in cancer, the roles of EV-related miRNAs and their potential applications in monitoring tools and therapeutic approaches are still under investigation.

In this report, we describe for the first time a comprehensive smRNA profile from the EVs of LM patient CSF, as obtained via an unbiased polyadenylation-based smRNA library construction procedure and subsequent NGS analysis. We performed deep sequencing on a subpopulation of relatively well-characterized smRNAs found in CSF EVs from LM patients and healthy control donors (HCs). The significance of biased expression of smRNA subpopulations was extensively validated using various biochemical methods. Moreover, the functionality of miR-21, which was found to be the most essential among the LM CSF EV-relevant smRNAs, was verified using a newly developed multipurpose lentivirus-based miR-21 monitoring system and physiological cell-based approaches. Finally, we discuss the potential roles of miR-21 and other essential smRNAs in the progression of LM and their usefulness for diagnosing LM.

## 2. Results

### 2.1. Biochemical and Molecular Characterization of EVs from CSF of LM Patients

Characteristics of the 19 LM patients and 16 HCs are summarized in Table 1 and Table S2.

**Figure 1:**
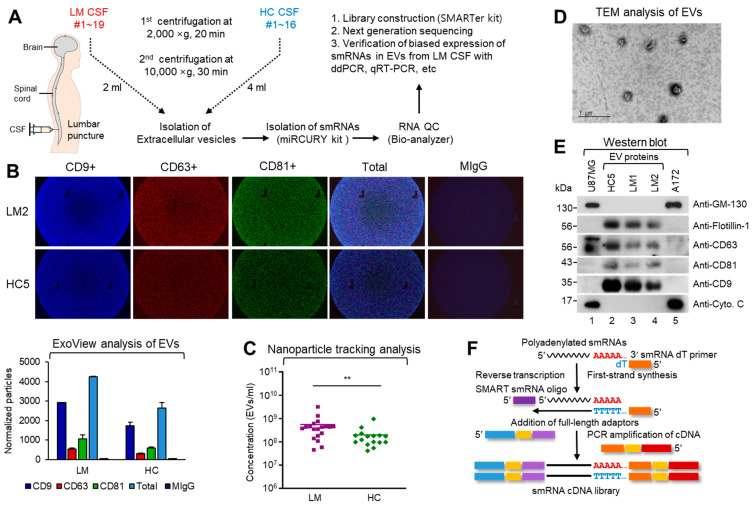
Biochemical and molecular characterization of EVs from CSF of LM patients. (**A**) Brief schematic flowchart and analytical procedure for patient sample collection, smRNA preparation, library construction, NGS, and biochemical analysis of EVs from CSF of LMs and HCs. CSF samples were obtained from patients harboring leptomeningeal metastasis, from intraventricular puncture (*n* = 12), cisternal puncture (*n* = 6) or lumbar puncture (*n* = 17). (**B**) The relative expression of EV specific markers was measured using ExoView R100 platform and Tetraspanin kit. CSF samples were incubated with the mixture of CD9-, CD63-, and CD81-capture antibodies on the Tetraspanin chip. After washing with washing buffer, the fluorescence images of chips were obtained with the confocal microscopy-based camera. Each antibody conjugated particles in 50-200 nm size were measured with the ExoView built-in software (bottom; bar graphs). Mouse IgG (MIgG) was used for negative control. The data represent the mean values of three independent experiments (*n* = 3) and error bars in the graph represent ± standard deviation. (**C**) Nanoparticle tracking analysis (NTA) revealed the difference in the EV concentrations between LM (purple rectangle, *n* = 19) and HC (green rhombus, *n* = 16). NTA was repeated 3 times per sample and an average value was provided (*n* = 3, two-tailed *t*-test, ** *p* < 0.01). (**D**) Transmission electron microscopy (TEM) shows bi-membranous vesicles of purified CSF EVs. scale bar: 1 µm. (**E**) Western blot analysis of EV markers (Flotillin-1, CD63, CD81 and CD9) and cellular proteins (GM-130 and cytochrome C in U87MG and A172 glioma cells) in HC and LM CSF EVs. (**F**) Schematic representation cDNA synthesis procedure of SMARTer smRNA-Seq method. Priming of a polyadenylated smRNA by a 3′ smRNA dT primer is followed by the synthesis of the first-strand cDNA by a MMLV-derived reverse transcriptase (RT). Extension and template switching then allows for a complete single stranded cDNA. PCR amplification allows for the addition of library adaptors and the synthesis of second-strand cDNA.

The mean age of all patients was 59.97 years (range, 36–73 years). Overall, 19 patients were female and 16 were male. Non-small cell lung cancer (NSCLC) was the most frequent primary cancer type among LM patients except LM8, 17, and 19 (breast cancer). To identify potential diagnostic small RNA (smRNA) biomarkers in LM patient CSF EVs, we first optimized a procedure for isolating EVs from a minimal amount of CSF (Figure 1A). All sample preparations and analyses were performed in accordance with our recently published work [8]. EVs were isolated from 2 mL (LM patients) or 4 mL (HCs) of CSF, and then the total RNAs were purified and further analyzed via NGS. As shown in Figure 1B, the presence and characteristics of the EVs were first verified using ExoView Tetraspanin Chip on the LM or HC CSF samples [8]. Beads bearing CD9/CD63/CD81 antibody-captured EVs were measured for their mean fluorescence intensity. Similar fluorescence patterns were obtained between LM patients and HCs, indicating that EVs were present in both LM and HC CSFs (Figure 1B, bottom).

After removing cellular components and fragments, we measured the concentrations and sizes of the presumed EVs from LM CSF by nanoparticle tracking analysis (NTA) using a NanoSight NS300 (for details, see Supplementary Materials and Methods; Figure 1C and Table S1). Nineteen LM patients exhibited a relatively high mean EV concentrations compared with 16 HCs (*p* = 0.004) which showed a similar pattern as we previously reported [8].

The EVs were isolated from LM CSF using a miRCURY exosome isolation kit (Figure 1D), and the purified EV pellets were used for western blotting (WB) to identify well-known EV markers [11,21,22,23,24]. As shown in Figure 1E and Figure S1, the representative EV markers, flotillin-1, CD63, CD81, and CD9, were clearly detected in HC and LM CSFs, and their expression patterns were similar between the groups. This confirmed that our EVs possessed biochemical characteristics similar to those reported previously. The absence of GM-130 (a Golgi marker) and cytochrome C (Cyto. C, a mitochondrial marker) excluded potential contamination with cellular vesicular structures, such as those from the Golgi and mitochondria [8].

Next, we sought to isolate total RNA from the EVs, as this would allow us to use NGS to analyze smRNA profiles. Total RNAs were isolated with a miRCURY RNA isolation kit and the criterion was evaluated based on the RNA size in the Bioanalyzer and the total amount of RNA in RiboGreen (Figure S1) [13].

For quality control during smRNA library construction, more than 10 commercially available RNA isolation kits were tested in the initial step. However, only the miRCURY RNA Isolation Kit-Cell & Plant (#300110, Exiqon; Qiagen, Hilden, Germany) was effective for RNA sequencing quality control and further library construction. As shown in Figure S1, smRNAs derived from HC and LM CSF-isolated EVs were analyzed using an Agilent Bioanalyzer with smRNA chips and showed relatively fair smRNA profiles for both HC and LM EVs. When we set out to construct the smRNA library, we tested numerous kits but obtained successful results only using the SMARTer smRNA-Seq Kit for Illumina (Takara Bio Inc., Shiga, Japan), which involves polyadenylation-mediated cDNA amplification of smRNA (Figure 1F). We then subjected the EV smRNA obtained from two healthy controls (HC1 and HC2) and eight LM patients (LM1-8) to smRNA NGS, which yielded averages of 6.4 million reads and 13.2 million reads, respectively. Uniquely clustered reads were then sequentially aligned to reference genome, miRBase v21, and the non-coding RNA database, Rfam 9.1, to identify known miRNAs and other types of smRNA subpopulations [24,25,26].

### 2.2. Essential Subpopulation of smRNAs Show Biased Expression Patterns in LM CSF EVs

**Figure 2:**
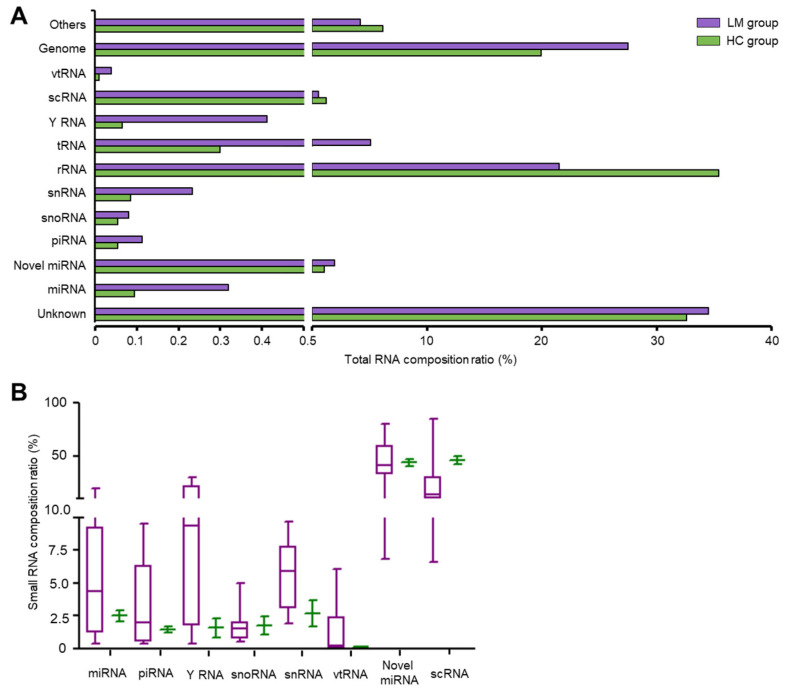
The proportion of smRNA subtypes showed biased expression between CSF EVs from LM and healthy control (HC). (**A**) smRNA sequencing data were processed for 10 types of general sm RNAs subclasses including genome and unknown population in RNA central database. Bar graph showed the average percentage of each smRNA subtype. (**B**) After removing reads mapped to rRNA, tRNA, and others, eight subtypes (miRNA, piRNA, Y RNA, snoRNA, snRNA, vtRNA, novel miRNA, and scRNA) of biologically intriguing smRNAs were processed. Boxplot showed the median percentage value and interquartile range of each smRNA subtypes. (LM, purple box, *n* = 8; HC, green bar, *n* = 2).

From among the annotated smRNA populations, we identified and focused on well-known classes that were present and asymmetrically distributed in HC versus LM CSF EVs. The most abundant housekeeping RNAs and contaminating mRNA fragments were excluded from the comparison and further analysis. We determined the relative distributions of the ten most abundant classes between HC and LM EVs, which corresponded to 33.1% of all aligned reads. These RNA classes included miRNA, piRNA, Y RNA, snoRNA, snRNA, vtRNA, novel miRNA, and scRNA (Figure 2).

The remaining aligned reads of EVs represented rRNA, tRNA, etc. All of the sorted and identified RNA classes exhibited differential distribution between HC and LM. Interestingly, as shown in Figure 2B, all of these smRNA subpopulations were relatively enriched in EVs from LM CSF compared to HC.

### 2.3. Analysis of Relative Expression Profiles and Related Cellular Pathways of miRNA in EVs from LM Patient CSF

**Figure 3:**
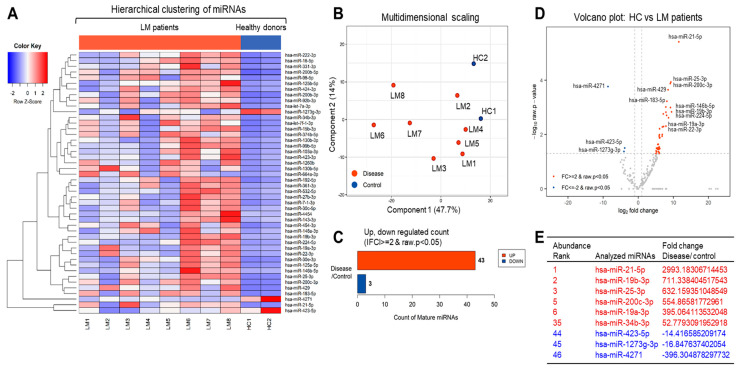
Distinct expression of miRNAs in EVs from LM patient CSF. Analysis of relative miRNA expression profile in EVs extracted from CSF of LMs and HCs. Hierarchical clustering analysis of significantly expressed miRNA was visualized via (**A**) heatmap showing z score of extravesicular miRNA from HC (*n* = 2) and patients with LM (*n* = 8) with 43 upregulated (red) and 3 downregulated (blue) miRNAs. (**B**) Multidimensional scaling (MDS) map of HC and LM was generated with proximity (calculated in Euclidean distance) indicating similarity, and (**C**) count of up- and downregulated mature miRNAs were found by fold change and *p*-value. (**D**) Volcano plot shows differentially expressed miRNA in HC and LM patients with the x-axis showing log2 fold-change and y-axis showing −log10 of the *p*-value from LM versus HC miRNA expression counts. (**E**) Table displays the fold change of 6 upregulated and 3 downregulated miRNAs in LM compared to HC ranked in the order of abundance.

To profile the EV miRNAs, we obtained approximately 3.7 million to 24.3 million raw reads using Illumina HiSeq. The raw reads of EV miRNAs from LM patient CSF were preprocessed, analyzed with miRDeep2, and then trimmed for adapter sequences [27,28]. Differentially expressed miRNAs between HC and LM CSF EVs were determined by selecting those with |fold change| ≥ 2 and *p*-value < 0.05. A total of 46 significantly differentially expressed miRNAs were identified, and hierarchical clustering showed that the miRNA expression profile of LM EVs was markedly distinct from that of HC (Figure 3 and Figure S2).

**Figure 4:**
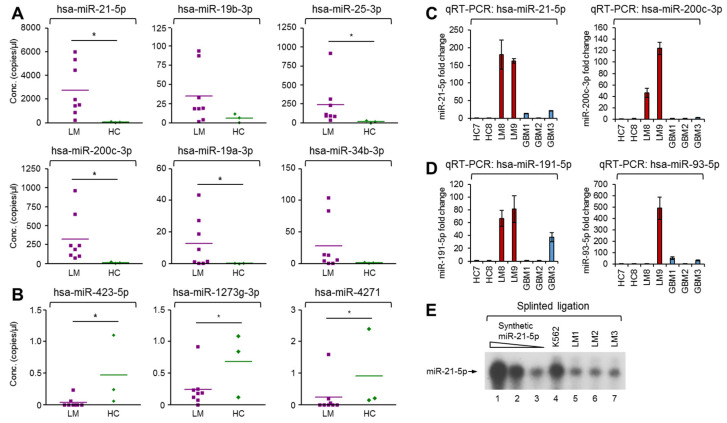
Biochemical verification of biased miRNA expression in EVs derived from LM and HC by ddPCR. (**A**) 6 upregulated miRNAs were analyzed by ddPCR (ddPCR Supermix for Probes; hsa-miR-21-5p, hsa-miR-19b-3p, hsa-miR-25-3p, hsa-miR-200c-3p, hsa-miR-19a-3p, and hsa-miR-34b-3p) in EVs from LM (purple rectangle, *n* = 8) vs HC (green rhombus, *n* = 3). (**B**) 3 downregulated miRNAs were analyzed by ddPCR (QX200 ddPCR EvaGreen Supermix; hsa-miR-423-5p, hsa-miR-1273g-3p, and hsa-miR-4271) in EVs from LM (purple rectangle, *n* = 8) vs HC (green rhombus, *n* = 3) (Mann-Whitney U test, * *p* < 0.05). (**C**) 2 upregulated miRNAs were analyzed by qRT-PCR (TaqMan Advanced miRNA Assay probes; miR-21-5p and miR-200c-3p) in CSF EVs from 2 LMs (LM8 and LM9), 2 HCs (HC7 and HC8) and 3 glioblastoma multiforme patients (GBM1-3). (**D**) 2 upregulated miRNAs were analyzed by qRT-PCR (TaqMan Advanced miRNA Assay probes; hsa-miR-191-5p and hsa-miR-93-5p) in CSF EVs from 2 LMs (LM8 and LM9), 2 HCs (HC7 and HC8), and 3 glioblastoma multiforme patients (GBM1-3). The data represent the mean values of three independent experiments (*n* = 3) and error bars in the graph represent ± standard deviation. (**E**) Splinted ligation was performed with [^32^P] 5′-end-labeled oligonucleotide probe specific for mature miR-21-5p as described in Supplementary Materials and Methods. The experiments were repeated at least three times with similar results. The data shown in panel (**E**) is a representative image.

Next, we validated the nine differentially expressed miRNAs using droplet digital polymerase chain reaction (ddPCR) [29]. We selected hsa-miR-21-5p, hsa-miR-19b-3p, hsa-miR-25-3p, hsa-miR-200c-3p, hsa-miR-19a-3p, and hsa-miR-34b-3p for validation of the biased expression between LM and HC. As expected, these miRNA molecules were significantly enriched in LM CSF EVs compared with HC (Figure 4A). In contrast, hsa-miR-423-5p, hsa-miR-1273g-3p, and hsa-miR-4271, which were found to be significantly downregulated in LM CSF EVs, were validated as being significantly downregulated in our ddPCR analysis (Figure 4B).

As shown in Figure 3E, hsa-miR-21-5p was ranked first in relative expression. We thus selected miR-21 for further analysis. This expression was validated using and conventional TaqMan probe-based real-time reverse transcription PCR (qRT-PCR). hsa-miR-200c-3p, which was the fifth-ranked miRNA in LM CSF EVs, was also selected for further analysis since it is known to play important roles in the metastasis and mobilization of cancer cells. As shown in Figure 4C, qRT-PCR result showed more than hundred folds elevation of both miRNAs in EVs from LM CSF which was already analyzed as significantly upregulated by NGS. The biased expression of miR-21 and miR-200c in LM CSF EVs was particularly notable since the expression levels were found to be relatively lower in CSF EVs from glioblastoma multiforme (GBM) patients than those from LM CSF. These data suggest that some yet-unknown driving mechanism governing the biased expression of both miRNAs is more potent in LM CSF EVs than in GBM CSF EVs. This may explain the poorer outcome of LM.

Out of the 46 miRNAs classified herein, additional essential miRNAs, such as hsa-miR-191-5p and hsa-miR-93-5p, also showed higher expression in LM CSF EVs than HC and GBM EVs (Figure 4D). In contrast, hsa-miR-204-5p and hsa-let-7b-5p, which are well-known to play tumor-suppressive roles [30], showed severe downregulation in LM CSF EVs compared with GBM CSF EVs (Figure S3).

Furthermore, we visualized the expression of miR-21-5p using a well-established conventional biochemical approach. Splinted ligation demonstrated that our ddPCR and qRT-PCR results were not caused by non-specific amplification of unwanted RNA species. As shown in Figure 4E and Figure S16, ligated bands of LM EV-derived and cellular miR-21-5p (from K562 cells) migrated to the same point as the synthetic miR-21-5p control. Moreover, similarly high and biased expression of miR-21-5p was observed in additional LM patient CSF EVs (Figure S3A), as assessed by ddPCR analysis. Our NGS (comprehensive high-throughput) and standard biochemical approaches clearly demonstrated that miR-21 is present in the EVs from CSF of LM patients. Collectively, these results suggested that the biased existence of specific population of miRNAs may contribute to LM progression.

**Figure 5:**
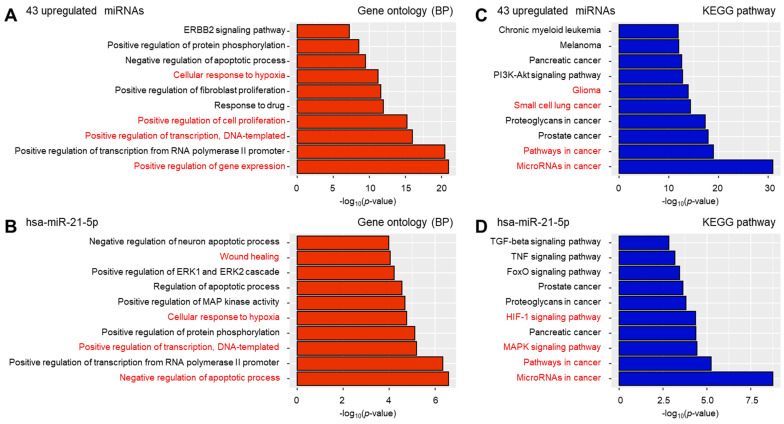
Analysis of the miRNA target gene enriched pathway. The experimentally validated target genes of 43 upregulated miRNAs (**A**,**C**) or hsa-miR-21-5p (**B**,**D**) were searched from miRTargetLink Human database. The target gene enriched pathways were computed from the Database for Annotation, Visualization and Integrated Discovery (DAVID). Bar graph depicted the top 10 highly enriched and biologically relevant pathways on Gene Ontology (GO) and Kyoto Encyclopedia of Genes and Genomes (KEGG) pathway. BP, biological processes.

To investigate the predicted functions of the miRNAs that were differentially expressed in LM EVs and HC EVs, we used the Database for Annotation, Visualization and Integrated Discovery (DAVID) functional annotation tool to perform Gene Ontology (GO) analysis. The significantly enriched GO terms included biological processes and molecular functions (Figure 5).

The 43 miRNAs upregulated in LM CSF EVs represented processes such as cellular response to hypoxia, positive regulation of cell proliferation, positive regulation of transcription, and positive regulation of gene expression in the biological process category. The selected miRNA, miR-21-5p, is involved in wound healing, cellular response to hypoxia, positive regulation of transcription, and negative regulation of apoptotic process.

We also used a Kyoto Encyclopedia of Genes and Genomes (KEGG) pathway enrichment analysis to examine the signaling pathways associated with each of the 43 miRNAs found to be differentially expressed in LM CSF EVs versus HC. Our KEGG analysis revealed that the pathways of glioma, small cell lung cancer, pathways in cancer, and miRNAs in cancer were particularly relevant. For miR-21-5p, the pathways of HIF-1 signaling, MAPK signaling, cancer and miRNAs in cancer were found to be significant.

### 2.4. miRNA Sensor-Based Investigation of miR-21 Functionality in EVs from LM Patient CSF

**Figure 6:**
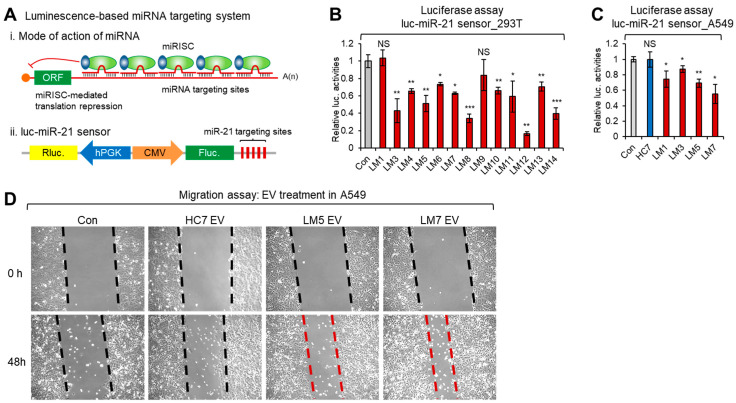
miRNA sensor and cellular migration-based investigation of extravesicular miR-21 functionality of LM patient CSF. (**A**) Mode of action of miRNA targeting (i), and schematic depiction of luminescence-based miR-21 sensor system (ii). miRISC; miRNA-induced silencing complex. (**B**,**C**) Relative firefly luciferase activities showed inverse correlation with functional miR-21 expression level in CSFs (**B**) and EVs (**C**), which binds to the miRNA targeting sites in 3′UTR of firefly luciferase in each luc-miR-21 sensor-bearing 293T (**B**) and NSCLC A549 (**C**) cells. *Renilla* luciferase activities were used in normalization of firefly luciferase activities. Data represent the mean values of three independent experiments (*n* = 3). Error bars in the graph represent ± standard deviation, and the *p*-values compare each LM CSF group to medium control (Con). * *p* < 0.05, ** *p* < 0.01, *** *p* < 0.001; NS, not significant. (**D**) Representative images of A549 migration assays after 48 h treatment with EVs from LM CSF (LM5 and LM7) and HC (HC7). Phase contrast microscopy images were taken with Axio Observer (100×, Zeiss).

To demonstrate whether the miRNA in the EVs from LM CSF could be physically incorporated into the miRNA-induced silencing complex (miRISC) and be functional within the miRNA machinery, we devised a lentivirus-based miR-21-sensing reporter. In this system, firefly luciferase is transcribed under the control of the cytomegalovirus (CMV) promoter and the translation of its mRNA is governed by five consecutive miR-21-targeting sites located in the 3′UTR. The devised system offers the ability to easily monitor the positive role of miR-21-containing LM CSF. The expression of firefly luciferase can be easily normalized using human phosphoglycerate kinase 1 (hPGK) promoter-driven *Renilla* luciferase activity in a dual luciferase assay (see Figure 6A). More details on this and other miRNA-monitoring sensors were described in our recent report [31]. In the present study, the binding of miR-21 to its target sites in the 3′UTR of the firefly luciferase mRNA repressed its translation, such that firefly luciferase activity was inversely correlated with the cellular level of miR-21 [31]. When we used the developed system to examine cellular expression in various cell lines, we found that miR-21 was highly expressed in NSCLC A549 cells and was barely detectable in 293T [31].

Next, to test the specific reactivity of luc-miR-21 sensor against LM CSF containing potentially functional miR-21s, we treated LM CSF directly onto luc-miR-21 sensor-bearing 293T cells. As shown in Figure 6B, in most cases, the LM CSF markedly inhibited the translation of firefly luciferase in luc-miR-21 sensor-bearing 293T cells. The physiological activity of LM CSF harboring miR-21 was further confirmed by directly applying isolated LM and HC CSF EVs to luc-miR-21 sensor-bearing A549 cells. As shown in Figure 6C, LM CSF EV inhibited the translation of firefly luciferase in luc-miR-21 sensor-bearing A549 cells, whereas HC CSF EVs did not.

These data collectively demonstrated that LM CSF and LM CSF EVs contain functional miR-21, indicating that this miRNA is physically incorporated into the cellular miRISC. This event can potentiate downstream cellular oncogenic signal cascades triggered by miR-21, which may induce LM malignancy among targeted recipient cells in patients.

### 2.5. Effects of miR-21-Containing LM CSF EVs on the Migratory Phenotype of NSCLC A549 Cells

To test whether miR-21 from LM CSF EVs can potentiate downstream cellular oncogenic signal cascades, we used a migration (wound-healing) assay. First, to elucidate the effect of miR-21 on the migration of A549 cells directly in vitro, we transfected the cells with synthetic miRNA mimics or negative control nucleotides and performed a migration assay. As shown in the Figure S4, the migratory phenotype of A549 cells was markedly enhanced at the edge of the scratch at 48 h in cells transfected with synthetic miR-21-5p, but not in those subjected to mock transfection or transfected with negative control nucleotides. Based on this observation, we next tested the effect of LM CSF EVs on NSCLC A549 cell motility. As shown in Figure 6D, Figures S13 and S17, the migratory phenotype of A549 cells was markedly enhanced at the edge of the scratch at 48 h in cells treated with LM CSF EVs, compared to untreated control cells or those treated with HC EVs. These results collectively suggested that EVs from LM CSF can promote the motility of NSCLC A549 cells in vitro, and these EVs contain a key regulator(s) of migration; thus, may be mediated by miR-21.

### 2.6. Biased Expression of Piwi-Interacting RNAs and Y RNAs in EVs from LM Patient CSF

**Figure 7:**
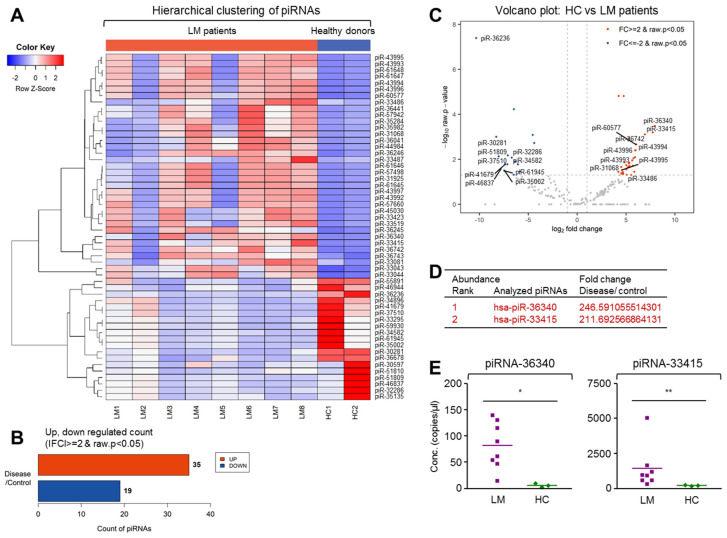
Distinct expression of Piwi-interacting RNAs in EVs from LM patient CSF. Analysis of relative Piwi-interacting RNA (piRNA) expression profile in EVs extracted from LMs and HCs. Hierarchical clustering analysis of significantly expressed piRNA was visualized via (**A**) heatmap showing z score of extravesicular piRNA from HC (*n* = 2) and patients with LM (*n* = 8) with 54 piRNA satisfying FC2 and raw *p*-value. (**B**) Count of up- and downregulated piRNA was found by fold change and raw *p*-value. (**C**) Volcano plot shows differentially expressed piRNA in HC and LM patients with the x-axis showing log2 fold-change and y-axis showing -log10 of the raw *p*-value from LM versus HC piRNA expression counts. (**D**) Table displays the fold change of two piRNA in LM compared to HC ranked in the order of abundance. (**E**) Level of the two piRNAs was confirmed in HC (*n* = 3) and LM EVs (*n* = 8) using ddPCR (piRNA-36340: Mann-Whitney U test, * *p* < 0.05; piRNA-33415: Unpaired *t*-test with Welch’s correction, ** *p* < 0.01).

Interestingly, NGS analysis of the LM CSF EVs revealed the biased expression of Piwi-interacting RNAs (piRNAs). piRNAs form RNA-protein complexes through interactions with Piwi-subfamily proteins. The piRNA complexes are mostly involved in the epi-genetic and posttranscriptional silencing of transposable elements [32]. As far as we know, this is the first report of piRNA expression in LM CSF EVs. As shown in Figure 7, hierarchical clustering revealed that piRNA expression could be clearly distinguished between LM CSF EVs and HC. Our analysis showed that 35 piRNAs were significantly upregulated in LM CSF EVs, while 19 were significantly downregulated. Our results suggested that piRNA could be a pathogenic index that could be used to discriminate LM. The analyzed LM samples showed similar clustering groups, whereas HC samples showed a very distinctive pattern (Figure S5). We further used ddPCR to validate two highly ranked piRNAs among the differentially expressed molecules. As shown in Figure 7E, hsa-piR-36340 and hsa-piR-33415 were significantly enriched in LM CSF EVs compared with HC.

**Figure 8:**
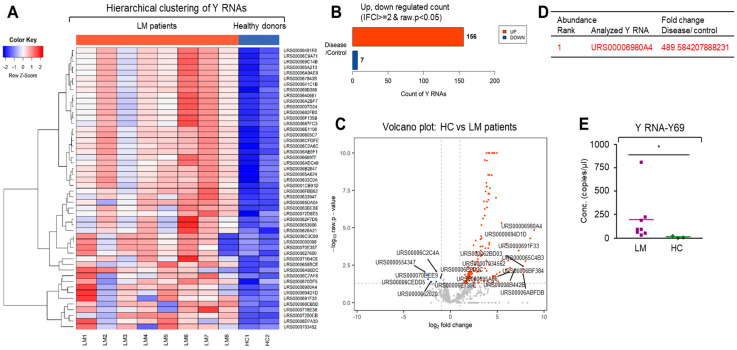
Distinct expression of Y RNAs in EVs from LM patient CSF. Analysis of relative Y RNA expression profile in EVs extracted from LMs and HCs. Hierarchical clustering analysis of significantly expressed Y RNA was visualized via (**A**) heatmap showing z score of extravesicular Y RNA from HC (*n* = 2) and patients with LM (*n* = 8) with 50 Y RNA that best satisfied FC2 value and adjusted *p*-value. (**B**) Count of up- and downregulated Y RNA was found by fold change and raw *p*-value. (**C**) Volcano plot shows differentially expressed Y RNA in HC and LM patients with the x-axis showing log2 fold-change and y-axis showing -log10 of the raw *p*-value from LM versus HC Y RNA expression counts. (**D**) Table displays the fold change of a Y RNA in LM compared to HC ranked in the order of abundance. (**E**) The upregulated Y RNA-Y69 expression was confirmed in HC (*n* = 3) and LM EVs (*n* = 8) using ddPCR (Mann-Whitney U test, * *p* < 0.05).

Another interesting smRNA species, Y RNAs, also showed markedly biased expression patterns between LM CSF EVs and HC. The members of this well-characterized smRNA subpopulation act as components of the Ro60 ribonucleoprotein particle, which is a target of autoimmune antibodies in patients with systemic lupus erythematosus [33]. Y RNAs are also necessary for DNA replication through their interactions with chromatin and initiation proteins [34]. In the LM CSF EVs, the number of upregulated Y RNAs (156) was greater than that seen for miRNA or piRNA, whereas only seven Y RNAs were downregulated. Our data suggested that Y RNA could be very useful as diagnostic biomarkers for LM. We used ddPCR to validate Y RNA-Y69, which was highly ranked as being differentially expressed in LM CSF EVs. As shown in Figure 8E, this confirmed that Y RNA-Y69 was significantly enriched in LM CSF EVs compared with HC CSF EVs.

### 2.7. Biased Expression of Essential Small RNA Subpopulations in EVs from LM Patient CSF: snRNA, snoRNA, vtRNA, Novel miRNA, and scRNA

We additionally identified meaningful and essential smRNA subpopulations that showed markedly biased expression in our NGS analysis of LM CSF EVs versus HC. As shown in Figures S10 and S11, the analyzed reads were annotated for identification of novel miRNAs, which were predicted from mature, star, and loop sequences according to the RNAfold algorithm miRDeep2 [28]. In contrast to the well-identified miRNA, the potential novel miRNA identified based on this analysis were largely downregulated (48, |fold change|≥ 2 and *p*-value < 0.05), with only nine exhibiting upregulation. This suggested that, except for the nine cases, most of the predicted novel miRNAs may play a role in LM progression, opposing the function of known miRNAs. Future work is warranted to examine whether these predicted novel miRNAs are expressed and functional. We used ddPCR to validate the highly ranked upregulated novel miRNA-973. As shown in Figure S10, novel miRNA-973 was significantly enriched in LM EVs compared with HC. The data indicated that at least the upregulated novel miRNAs are processed in the LM CSF EVs and thus may exert positive functions in LM pathogenesis.

Finally, we examined essential smRNAs, such as snRNA, snoRNA, vtRNA, and scRNA (Figures S6–S9, S11, and S12). We found that snRNA, snoRNA, and vtRNA were highly enriched in LM CSF EVs. Without exception, all of the identified snRNA, snoRNA, and vtRNA were detected to a much greater extent in LM CSF EVs compared to HC. We could speculate that the skewed distribution of snoRNAs in LM CSF EVs is likely a reflection of their biological function(s) in the cell, whereas the observed enrichment of several snoRNA fragments may be attributed to a miRNA-like cytoplasmic function. snRNA-200C70, vtRNA-6C57F3, and scRNA-F3729, which were found to be differentially expressed in LM CSF EVs versus HC, were checked by ddPCR. As shown in Figures S6, S9, and S12, they were significantly enriched in LM EVs compared with HC. This suggests that these smRNAs may contribute to LM pathogenesis.

## 3. Discussion

### 3.1. Comprehensive and Quantitative Analysis of Essential smRNA Subpopulation in EVs from LM patient CSF

LM is generally considered to be a late complication of solid tumors [3]. LM causes fatal cancer complications; thus its early diagnosis and monitoring are essential for effective treatment and improved disease prognosis [2]. To date, we lack any clear biomarkers that reflect the disease progression or molecular mechanisms of LM; this can be a major obstacle to finding effective treatments. Therefore, efforts to identify biomarkers for monitoring and investigating the mechanisms of the disease are critical to improve the prognosis of LM and explore new therapeutic options. In several studies on LM, CSF cytology and magnetic resonance imaging methods were used for diagnosis; however, these methods are highly dependent on the examiner and are limited by reader-to-reader variability – entailing a high risk for non-specific results [2,6]. Moreover, repeated lumbar punctures for CSF cytology are not favorable for patients [35]. The data from this study strongly indicate that analysis of smRNAs in CSF could be a promising approach for developing minimally invasive assays for LM detection.

In this study, we examined CSF smRNA levels in individual LM patients as potential markers for monitoring disease diagnosis. This is the first comprehensive and systemic analysis of the smRNA molecular profiling in a standard set of CSF samples from individual LM and non-LM patients. The identification of specific CSF smRNAs in this study suggests new ways to diagnose and monitor LM and may contribute to efforts to explore the mechanisms of LM.

The use of smRNA as LM biomarkers could offer several benefits. First, smRNAs, essentially miRNAs, are functional in the biological system, and thus could be easily applied for biochemical and physiological assays [13,20]. Here, we demonstrated that LM CSF EV miRNA are physiologically functional using a specific miR-21 biosensor and various cell-based assays. Second, smRNAs have sequence specificity and may be amplified with various biochemical assays, making them suitable for disease-specific diagnostics [20]. Third, smRNAs are useful because they are typically less than 200-bp in size, and thus do not need to be fragmented prior to library preparation [36,37]. Finally, storage conditions do not influence the quality or distribution of the recovered smRNAs [38]. Therefore, smRNA could be better biomarkers for the diagnosis of LM compared to macromolecules such as large RNA, DNA, proteins, etc.

Our approach and quantitative-qualitative analyses of the smRNAs from LM CSF EVs are superior to those of previous reports, as indicated by the following lines of evidence. First, in this study, we set up a small-scale (2–4 mL of CSF) pipeline for analyzing the entire small RNA transcriptome of CSF EVs. We successfully conducted RNA-sequencing of extravesicular smRNAs using 3′-end polyadenylation-based SMARTer smRNA-Seq commercial kit. We tested around 10 other kits for total RNA isolation that did not pass library quality control and/or were not successful for library construction. In addition, this protocol, which does not require laborious ultracentrifugation or large volumes of CSF, may be practical in the clinical setting.

Previous reports showed that conventional adaptor-based approaches yielded biased results for smRNA populations [37]. Compared to the conventional method, the polyadenylated method offers easier construction of the smRNA library, an improved success rate for library construction, and a decreased risk for bias due to the 5′ and 3′ end nucleotide composition. To our knowledge, this is the first report of the global identification of smRNAs from the LM CSF EVs of individual patients. In addition, our study utilized small-scale volume of CSF, unlike previous studies in which samples were pooled or required large volumes of CSF [13]. Yagi et al. previously performed NGS-based miRNA profiling of CSF exosomes. However, the authors used CSF from several healthy volunteers, did not analyze disease-related smRNAs, and examined only miRNAs. Here, we systemically analyzed the extravesicular smRNA profile at an individual level. In this way, we may achieve our goal of efficient personalized and diseased-oriented analysis of smRNA populations. In addition to relevant miRNAs, we first analyzed and identified other essential smRNA subpopulations, including piRNA, Y RNA, snRNA, snoRNA, and vtRNA, in the LM CSF EV context.

Second, the biased expression of LM CSF extravesicular smRNA was systemically, biochemically, and functionally validated through various biochemical and cellular approaches. Interestingly, we found that the potential LM pathogenesis-related miRNAs were highly upregulated in LM CSF EVs. Our study also revealed that the ratios of different types of extravesicular smRNAs differed between LM patients and HC. Furthermore, identification of new smRNA population will increase our knowledge of LM.

Finally, the presence of smRNAs separated and identified on a large scale was quantitatively confirmed using ddPCR (also see Figure S14 for normalized expression) as well as qRT-PCR. These validated smRNA species, which may be analyzed using simple and precise biochemistry, could potentially be used as cost-saving and efficient biomarkers. We urgently need new strategies for the molecular diagnosis and liquid biopsy of LM using simple and effective biochemical approaches.

### 3.2. Implication of miRNAs for LM Pathogenesis and as Essential Biomarkers for LM Diagnosis

Through the analysis of smRNA populations via a polyadenylation-mediated library construction method, we identified 46 miRNAs that showed meaningful differences between LM CSF EVs and HC. We biochemically validated the differential expression of hsa-miR-21-5p, hsa-miR-19b-3p, hsa-miR-25-3p, hsa-miR-200c-3p, hsa-miR-19a-3p, and hsa-miR-34b-3p, which were significantly higher in LM patients than in HCs. The top-scoring miRNA, miR-21, is intriguing since it has been suggested as a critical biomarker of various cancers and is generally considered to act as an oncogene by negatively regulating various tumor-suppressive target mRNAs [39]. For example, miR-21 plays significant roles in central nervous system (CNS)-related tumors [40]. Our group and others previously showed that miR-21 expression is tightly correlated with the malignancy of glioma [31,41], and we have recently shown that miR-21 exhibited significant biased expression after chemotherapeutic treatment [8]. Thus, the previous and present findings suggest that miR-21 could be an essential biomarker for the treatment response of LM. Moreover, our experimental results suggest that miRNAs, especially miR-21, are key components of LM pathogenesis and characterization.

We systemically validated the function of extravesicular miR-21 in LM CSF via our newly developed miR-21 sensor [31]. Using this sensitive system, we were confident that LM CSF contains functional miRNAs, not just meaningless smRNA fragments. This was also confirmed with isolated EVs. Our data indicated that LM CSF EVs, which contain higher level of functional miR-21, positively affected the migratory phenotype of NSCLC A549 cells, whereas HC CSF EV did not.

Another top-scoring miRNA, miR-19b, is known to enhance proliferation and apoptosis resistance via the EGFR signaling pathway by targeting PP2A and BIM in non-small cell lung cancer [42]. miR-200c, which is a member of the miR-200 family, regulates epithelial-mesenchymal transition by downregulating ZEB1/2 and upregulating E-cadherin [43]. miR-200c also has important functions in proliferation, invasion, and metastasis [44]. Furthermore, miR-200c is a well-established prognostic and diagnostic marker in different cancer types. Another study investigating serum miRNAs as cancer biomarkers showed that miR-200c is associated with NSCLC, suggesting that it could potentially be useful for diagnosis [45].

Our GO analyses showed that the target genes of 43 miRNAs, including top-scored miR-21, were involved in regulating cell migration and cell differentiation. Based on the existing literature and our experimental results, we speculate that miR-21 containing 43 miRNAs may have a critical function for LM pathogenesis [8,17]. The increase of miRNA expression may activate proliferation, invasion, and migration, which are closely related to cancer metastasis mechanisms; thus promoting the occurrence of LM. We also performed KEGG pathway analysis for the target genes of the 43 miRNAs and found that they were involved in the pathways related to glioma, small cell lung cancer, pathways in cancer, and miRNAs in cancer. Many of these pathways have close connections to CNS metastasis, and abnormal signaling is likely to play a crucial role in the development of LM. Accumulating evidence has demonstrated that the activation of MAPK, PI3K-Akt, and HIF-1 signaling pathways are important for cancer progression. Further molecular investigations are needed and such work will likely provide new perspectives on the mechanisms of LM and suggest new intervention methods in LM treatment.

### 3.3. Implication of smRNA Subpopulations for LM Pathogenesis and as Potential Biomarkers for LM Diagnosis: piRNA, Y RNA, and other smRNAs

One of the smRNAs that showed significantly biased expression in our NGS analysis was piRNA. The piRNAs comprise the largest class of smRNA molecules expressed in animal cells. They are around 26–31-nt long, and form RNA-protein complexes through interactions with Piwi-subfamily proteins [46]. The formed piRNA complexes are mostly involved in the epigenetic and posttranscriptional silencing of transposable elements, but can also contribute to regulating other genetic elements in germline cells [32]. Consistent with our data, a growing number of studies have shown that piRNA and Piwi proteins, which are abnormally expressed in various cancers, may serve as novel biomarkers and therapeutic targets. However, the functions of piRNAs in cancer and their underlying mechanisms are not fully understood [47]. As shown in Figure 7, hierarchical clustering of piRNA expression yielded clearly different patterns in LM CSF EVs compared with HC. Thirty-five piRNAs were significantly upregulated and 19 piRNAs were significantly downregulated in LM CSF EVs versus HC. Notably, our hierarchical clustering suggested that piRNAs could be used as an index to discriminate LM. To our knowledge, this is the first study to show that biased expression piRNA in LM CSF EVs. Therefore, the essential role of piRNA for the LM complication should be pursued in the near future.

Another intriguing smRNA subpopulation that was highly upregulated in EVs from LM CSF is Y RNAs. Y RNAs are highly conserved non-coding RNAs of ~100-nt in size. These smRNAs are components of the Ro60 ribonucleoprotein particle, which is a target of autoimmune antibodies in patients with systemic lupus erythematosus. These RNAs are involved in various basic intracellular processes, such as DNA replication and RNA quality control [33]. Several studies have reported the abundant presence of Y RNAs in cell culture EV and body fluids. However, relatively little work has focused on the function and biomarker potential of extracellular Y RNAs. Notably, Y RNAs are overexpressed in some human tumors and required for cell proliferation, and miRNA-sized breakdown products of Y RNAs may be involved in other unknown pathological conditions [34]. As shown in Figure 8, hierarchical clustering showed that Y RNA expression is also distinctive in LM CSF EVs compared with HC. Our results revealed that 156 Y RNAs were significantly upregulated in LM CSF EVs, but only seven Y RNAs were significantly downregulated. The essential role of Y RNA for the LM complication also should be pursued in near future.

snoRNAs are a class of smRNA molecules that primarily guide chemical modifications of other RNAs, mainly rRNAs, tRNAs, and snRNAs. snoRNAs can function as miRNAs. It has been shown that human ACA45 snoRNA can be processed into a 21-nt long mature miRNA by Dicer [48]. Bioinformatic analyses have revealed that putatively snoRNA-derived, miRNA-like fragments occur in different organisms [49]. Mutations and aberrant expression of snoRNAs have been reported in cell transformation, tumorigenesis, and metastasis, indicating that snoRNAs may serve as biomarkers and/or therapeutic targets for cancer [50]. During LM development, aberrant expression of snoRNA may contribute to LM-related complications. Alternatively, the miRNA-like activity of snoRNA may affect recipient cells upon LM CSF EV delivery.

snRNAs represent a class of small RNA molecules that are found within the splicing speckles and Cajal bodies of the cell nucleus in eukaryotic cells. snRNA average ~150-nt in length, and their primary function is in the processing of pre-messenger RNA (hnRNA) in the nucleus and the U1 spliceosomal RNA is recurrently mutated in multiple cancers [51,52].

A major obstacle in cancer treatment is the development of chemoresistance, and vtRNAs are known to play a role in this phenomenon. vtRNAs may facilitate the export of certain chemotherapeutic drugs through binding-site specific interactions. In addition, recent studies suggest that vtRNAs may inhibit drug activity through interfering with drug target sites; thus conferring drug resistance during chemotherapy of LM [53].

With the exception of miRNAs, the smRNA subclasses have limited biochemical analysis tools and functional assays, making it difficult to functionally verify their cellular roles in LM. Furthermore, the lack of an animal model for LM further complicates the research. In the near future, we can hope that new proper model systems will be developed to facilitate the study of LM.

## 4. Materials and Methods

### 4.1. Collection of Clinical Samples and Preparatory Process

CSF samples were collected after approval of Institutional Review Board in National Cancer Center, Korea (NCC2014-0135) in accordance with the ethical guidelines outlined in the Declaration of Helsinki. The informed consent was obtained from all patients. CSF samples were obtained from each patient before the lumbar, intraventricular, and cisternal puncture. Obtained CSF was centrifuged within 1 h at 2000× *g* for 20 min for removing cells and cellular debris at room temperature. After first centrifugation, CSFs were further centrifuged at 10,000× *g* for 30 min and kept frozen at −80 °C.

### 4.2. ExoView Analysis of EVs in CSFs

The physical and biological properties of EVs in CSF samples were characterized by using ExoView R-100 (NanoView Biosciences, Boston, MA, USA) and ExoView Tetraspanin kits (NanoView Biosciences) including anti-CD81, anti-CD63 and anti-CD9 immobilized chips, labeling agents, washing solutions (solution A and B) and blocking agent (NanoView Biosciences). The three-capture antibody spots in each tetraspanin case were arrayed in one chip thus, average and standard deviation could be measured in one chip. Briefly, the 35 μL of diluted sample with solution A was dropped on the ExoView Tetraspanin chip and incubated overnight (16 h) at room temperature (RT). After the incubation process, the sample loaded chip was washed by 1 mL of solution A for 3 min and this was repeated by three times. Subsequently, the EVs on the chip were labeled by using 250 μL of a mixture of anti-CD81/Alexa Fluor 555 (green), anti-CD63/Alexa Fluor 647 (red), and anti-CD9/Alexa Fluor 488 (blue) and incubated for 1 h at RT to measure the colocalization of tetraspanin on the surface of EVs. In this case, the fluorescein-labeled antibody was diluted in mixture of solution A and blocking solution with 1:600. Finally, the chip was rinsed by 1 ml of solution A and B and dried at RT. The EV captured chip was scanned by ExoView R-100 via nScan software (NanoView Biosciences) and data were analyzed through NanoViewer 2.9 software (NanoView Biosciences).

### 4.3. Isolation of EVs from LM Patient CSFs

EVs were isolated from CSF of LM and HC individuals. The CSF samples were centrifuged twice and the cleared samples were used for the isolation of EVs with miRCURY Exosome Cell/Urine/CSF Kit (#300102, Exiqon) according to the manufacturer’s instructions. In brief, CSF sample gently mixed Precipitation Buffer B, then the mixtures were vortexed and incubated for 60 min at 2–8 °C for precipitating exosome pellet. After centrifugation at 10,000× *g* for 30 min at 20 °C, then the supernatant was removed.

### 4.4. EV RNA Extraction and Measurement

EV RNA extraction was performed using the miRCURY RNA Isolation Kit - Cell & Plant (#300110, Exiqon) following the manufacturer’s instructions. In brief, exosome pellet was lysed with the provided lysis solution supplemented with 96–100% ethanol, and the mixture was loaded to the column. The EV RNA was washed and eluted with 15–50 µL of RNase-free water. The extracted RNA concentration was calculated by Quant-IT RiboGreen (Invitrogen; Thermo Fisher Scientific, Waltham, MA, USA). RNA size was confirmed using Agilent RNA 6000 Pico Kit and Small RNA Kit on Agilent 2100 Bioanalyzer (Agilent Technologies, Waldbronn, Germany).

### 4.5. Small RNA Library Construction and Sequencing

The 10 ng of RNA isolated from each sample was used to construct sequencing libraries with the SMARTer smRNA-Seq Kit for Illumina (Takara Bio Inc.), following the manufacturer’s protocol. Input RNA was first polyadenylated in order to provide a priming sequence for an oligo(dT) primer. cDNA synthesis was primed by the 3′ smRNA dT Primer, which incorporates an adapter sequence at the 5′ end of each first-strand cDNA molecule. In the template-switching step, PrimeScript Reverse Transcriptase utilized the SMART smRNA Oligo as a template for the addition of a second adapter sequence to the 3′ end of each first-strand cDNA molecule. In the next step, full-length Illumina adapters (including index sequences for sample multiplexing) were added during PCR amplification. The forward PCR primer was then bound to the sequence added by the SMART smRNA Oligo, while the reverse PCR primer was bound to the sequence added by the 3′ smRNA dT Primer. The amplified libraries were purified from 6% Novex TBE-PAGE gels (Thermo-Fisher Scientific) to excise the fraction over 138-bp (over than 18-bp of cDNA plus 120-bp of adaptor). The resulting library cDNA included sequences required for clustering on an Illumina flow cell. The libraries were gel purified, and validated by checking the size, purity, and concentration on the Agilent 2100 Bioanalyzer. The libraries were then quantified using qPCR according to the qPCR quantification protocol guide (KAPA Library Quantification kits for Illumina Sequencing platforms; Roche, Basel, Switzerland) and qualified using the TapeStation D1000 ScreenTape (Agilent Technologies). The libraries were pooled in equimolar amounts, and sequenced on an Illumina HiSeq 2500 (Illumina, San Diego, CA, USA) instrument to generate 101 base reads. Image decomposition and quality values calculation were performed using the modules of the Illumina pipeline. Adapter trimming was assessed using the Cutadapt program.

### 4.6. Data Analysis of smRNA Sequencing

Uniquely clustered reads were then sequentially aligned to reference genome, miRBase v21 and non-coding RNA database Rfam 9.1 to identify known miRNAs and other type of smRNAs. Hierarchical cluster heatmaps, dendrograms, volcano plots, and multidimensional scaling (MDS) of extracted EV smRNA were performed by Macrogen (Seoul, Korea). Various hierarchical clustering analysis was performed using complete linkage and Euclidean distance as measures of similarity in differentially expressed patterns of the smRNA with the criteria of |fold change|≥ 2 and *p*-value < 0.05. Distance matrices were processed by MDS to obtain a dimensionally reduced map of gene coordinates with distance between plots as a measure of similarity. All data analysis and visualization were performed using R 3.6.3 (www.r-project.org) (Table S4).

### 4.7. Synthesis of smRNA cDNA and Droplet Digital PCR (ddPCR)

Approximately 2 ng of purified total EV RNA was reverse transcribed to generate cDNA with TaqMan Advanced miRNA cDNA Synthesis Kit (A28007, Applied Biosystems, Foster City, CA, USA) according to the manufacturer’s instruction. Four µL of cDNA was mixed ddPCR Supermix for Probes (No dUTP, Bio-Rad Laboratories, Inc., Hercules, CA, USA) and TaqMan Advanced miRNA Assay probes (Applied Biosystems; hsa-miR-21-5p, hsa-miR-19-3p, hsa-miR-25-3p, hsa-miR-200c-3p, hsa-miR-19a-3p, and hsa-miR-34b-3p). Four μL of cDNA was mixed with the QX200 ddPCR EvaGreen Supermix (Bio-Rad Laboratories, Inc.) and oligomers for the following smRNAs; hsa-miR-423-5p, hsa-miR-1273g-3p, hsa-miR-4271, piRNA-33415, piRNA-36340, Y RNA-Y69, snRNA-200C70, vtRNA-6C57F3, novel miRNA-973, and scRNA-F3729 (Table S3). Each 20 µL of reaction mixture was mixed with 70 µL of droplet generation oil and partitioned into up to 20,000 nL-sized droplets by a QX299 droplet generator (Bio-Rad Laboratories, Inc.). Final 40 µL droplet mixture was used for the PCR reaction following cycling protocol. ddPCR Supermix for probes; 95 °C for 5 min (DNA polymerase activation), followed by 40 cycles of 95 °C for 30 s (denaturation) and 55 °C for 1 min (annealing) followed by post-cycling steps of 98 °C for 10 min (enzyme inactivation) and an infinite 4 °C hold by Applied Biosystems 7900HT Sequence Detection System. QX200 ddPCR EvaGreen Supermix; 95 °C for 5 min (DNA polymerase activation), followed by 39 cycles of 95 °C for 30s (denaturation) and 60 °C for 1 min (annealing) followed by post-cycling steps of 1 cycle of 4 °C for 5 min, and 1 cycle of 90 °C for 5 min and an infinite 4 °C hold. Cycling between the temperatures was set to a ramp rate of 2.5 °C/s. Annealing temperature of each oligomer set was optimized in QX200 ddPCR EvaGreen Supermix-based PCR reaction. The amplified PCR product of the nucleic acid target in the droplets were quantified in the FAM channels using QC200 Droplet Reader (Bio-Rad Laboratories, Inc.) and analyzed using QuantaSoft v.1.7.4.0917 software (Bio-Rad Laboratories, Inc.). The concentration (smRNA copies/μL) value generated by QuantaSoft was converted to smRNA copies/nL of CSF. The Mann-Whitney U test and unpaired *t*-test with Welch’s correction were used to compare significant differences in each smRNA expression between two groups according to the result of normality test (Shapiro-Wilk test). * *p*-value < 0.05, ** *p*-value < 0.01.

### 4.8. The Gene Ontology of Biological Processes and the Kyoto Encyclopedia of Genes and Genomes Pathways

The putative target genes of miRNA were searched from miRTargetLink [54] providing a network algorithm based on the miRTarBase and experimentally validated interactions. The Gene Ontology (GO) of biological processes and the Kyoto Encyclopedia of Genes and Genomes (KEGG) pathways were computed using DAVID Bioinformatics [55]. 455 target genes of 43 miRNAs were identified from miRTargetLink database and gene set enrichment analysis was assessed.

### 4.9. Luciferase Assay of miR-21 Sensor-Bearing Cell Lines

Two luminescence-based miR-21 sensor-bearing cell lines (293T and A549) were established by lentiviral infection, and then harvested 16–20 h after treatment with designated CSFs or CSF EV concentrates. Cells were lysed with Passive Lysis Buffer (Promega, Madison, WI, USA), and the aliquot of lysates was analyzed by measuring luminescence signals with Dual-Luciferase Reporter Assay System (Promega) in a reader (SpectraMax L, Molecular Devices, San Jose, CA, USA). The miR-21 sensor signal from firefly luciferase was normalized with that from *Renilla* luciferase. The normalized quantification data was used in comparing the relative luciferase activities. Data are presented as the mean ± standard deviation determined from at least three independent experiments. Differences were assessed by two-tailed Student’s *t*-test using Excel software (Microsoft, Redmond, WA, USA). * *p*-value < 0.05, ** *p*-value < 0.01, *** *p*-value < 0.001; NS, not significant.

### 4.10. Migration Assay

Human NSCLC A549 cells were plated on 6-well plates at a density of 6 × 10^5^ per well and cultured up to 80–90% confluence for 2 days before scratching. Then 4–6 lines were scratched in each confluent monolayer with a sterile 200 µL tip. Dislodged cells were removed by washing with warm Dulbecco’s phosphate-buffered saline, and the remaining cells were replenished with fresh medium. A representative line with similar width were selected and photographed in each group at starting point, and then treated with concentrated EV dissolved medium or transfected with miR-21 mimics. After 24 h and 48 h, snapshots of the scratch were taken with the microscope (100×) (Axio Observer, Zeiss, Oberkochen, Germany).

## 5. Conclusions

We reveal herein the molecular profile of CSF extravesicular smRNAs that are potentially associated with LM pathogenesis. We successfully identified various LM-associated smRNA populations that showed significantly biased expression patterns in LM. Our extensive NGS analysis and relevant biochemical and cell-based validations demonstrated that miRNAs and the other analyzed smRNA subpopulations may be useful targets for the development of therapeutic and diagnostic strategies for LM. Further investigation is critically needed to address the potential of extravesicular smRNAs as a novel pharmacological target for LM and LM-related complications. Additional experiments and bioinformatic analyses may reveal novel means to explore the underlying mechanisms of LM and provide additional promising targets for treating LM patients.

## Figures and Tables

**Table 1:** Clinical characteristics of CSF samples and their applications in this study (*n* = 24).

Patients No.	Gender	Age	Patient Group	Primary Disease	Sample Site	Applications
LM1	Female	67	LM	NSCLC	Intraventricular	NGS, ddPCR, Luc, WB, SL
LM2	Female	67	LM	NSCLC	Lumbar	NGS, ddPCR, ExoView, WB, SL
LM3	Female	63	LM	NSCLC	Lumbar	NGS, ddPCR, Luc, SL
LM4	Male	44	LM	NSCLC	Lumbar	NGS, ddPCR, Luc
LM5	Female	54	LM	NSCLC	Lumbar	NGS, ddPCR, M.A., Luc
LM6	Male	54	LM	NSCLC	Lumbar	NGS, ddPCR, Luc
LM7	Male	69	LM	NSCLC	Lumbar	NGS, ddPCR, M.A., Luc
LM8	Female	36	LM	Breast cancer	Lumbar	NGS, ddPCR, ExoView qRT-PCR, Luc,
LM9	Female	55	LM	NSCLC	Intraventricular	ddPCR (E.V.), qRT-PCR, Luc
LM10	Male	62	LM	NSCLC	Intraventricular	ddPCR (E.V.), Luc
LM11	Male	65	LM	NSCLC	Intraventricular	Luc
LM12	Female	63	LM	NSCLC	Intraventricular	ddPCR (E.V.), Luc
LM13	Male	68	LM	NSCLC	Lumbar	Luc
LM14	Male	56	LM	NSCLC	Intraventricular	Luc
HC1	Female	61	Healthy control	Unruptured an	Cisternal	NGS
HC2	Female	60	Healthy control	Unruptured an	Cisternal	NGS, ddPCR
HC3	Male	50	Healthy control	Unruptured an	Cisternal	ddPCR
HC4	Female	73	Healthy control	Unruptured an	Cisternal	ddPCR
HC5	Female	45	Healthy control	Unruptured an	Lumbar	ExoView, WB, ddPCR (E.V.)
HC6	Male	69	Healthy control	Unruptured an	Lumbar	ExoView, M.A., Luc, ddPCR (E.V.)
HC7	Female	55	Healthy control	Unruptured an	Lumbar	qRT-PCR, M.A., Luc, ddPCR (E.V.)
HC8	Male	61	Healthy control	Unruptured an	Lumbar	qRT-PCR
HC9	Male	59	Healthy control	Unruptured an	Intraventricular	ddPCR (E.V.)
HC10	Male	40	Healthy control	Unruptured an	Intraventricular	ddPCR (E.V.)

LM, leptomeningeal metastasis; HC, healthy control; NSCLC, non-small cell lung cancer; Unruptured an, unruptured aneurysms; NGS, next generation sequencing; ddPCR, droplet digital PCR; qRT-PCR, real-time reverse transcription polymerase chain reaction; M.A., migration assay; Luc, luciferase assay; WB, western blot; SL, splinted ligation; E.V., external validation.

## Data Availability

The raw data that support the findings of this study are available from the corresponding authors upon reasonable request.

## Supplementary Materials

The following are available online at https://www.mdpi.com/2072-6694/13/2/209/s1, Figure S1: Bioanalyzer analysis of the size distribution of RNA from HC and LM CSF EVs; Figure S2: Hierarchical clustering dendrogram of miRNAs among LM patients; Figure S3: Biochemical verification of biased miRNA expression in EVs derived from LM and HC by ddPCR and qRT-PCR; Figure S4: Investigation of synthetic miR-21 functionality on cellular migratory phenotype of NSCLC A549 cells; Figure S5: Hierarchical clustering analysis of piRNAs and Y RNAs in EVs from LM patient CSF; Figure S6: Distinct expression of snRNAs in EVs from LM patient CSF; Figure S7: Distinct expression of snoRNAs in EVs from LM patient CSF; Figure S8: Hierarchical clustering analysis of snRNAs and snoRNAs in EVs from LM patient CSF; Figure S9: Distinct expression of vtRNAs in EVs from LM patient CSF; Figure S10: Distinct expression of novel miRNAs in EVs from LM patient CSF; Figure S11: Hierarchical clustering analysis of vtRNAs and novel miRNAs in EVs from LM patient CSF; Figure S12: Distinct expression of scRNAs in EVs from LM patient CSF; Figure S13: Western blot analysis of the validated target genes of miR-21 in LM CSF EV-treated A549 migration assay samples; Figure S14: Biochemical verification of normalized smRNA expression in EVs derived from LM and HC by ddPCR; Figure S15: Original images of western blot related with Figure 1E; Figure S16: Original image of splinted ligation related with Figure 4E; Figure S17: Original images of western blot related with Figure S13B; Table S1: Experimental raw data of the CSF samples in this study; Table S2: Clinical characteristics of additional CSF samples and their applications in this study (*n* = 11); Table S3: Oligonucleotides used in ddPCR analysis; Table S4: List of smRNA sequencing expression raw data by descending fold change and *p*-value.

## Abbreviations

EVs	Extracellular vesicles
LM	Leptomeningeal metastasis
NTA	Nanoparticle tracking analysis
CSF	Cerebrospinal fluid
ddPCR	Droplet digital polymerase chain reaction
NSCLC	Non-small cell lung cancer
smRNA	Small non-coding RNA
qRT-PCR	Real time reverse transcription PCR
GO	Gene Ontology
DAVID	Database for Annotation, Visualization and Integrated Discovery
KEGG	Kyoto Encyclopedia of Genes and Genomes
miRISC	miRNA-induced silencing complex
MDS	Multidimensional scaling
FC	Fold change
