# Supplementary material for: Molecular Signature of Extracellular Vesicular Small Non-Coding RNAs Derived from Cerebrospinal Fluid of Leptomeningeal Metastasis Patients: Functional Implication of miR-21 and Other Small RNAs in Cancer Malignancy
Source: Cancers (Basel). 2021 Jan 8;13(2):209. doi: 10.3390/cancers13020209 (PMC7828086; doi:10.3390/cancers13020209)
Supplement: Supplementary file 1 [file cancers-13-00209-s001.zip › cancers-13-00209-supplementary_materials.pdf]

cancers

MDPI

*Supplementary Materials*

# Molecular Signature of Extracellular Vesicular Small Non-Coding RNAs Derived from Cerebrospinal Fluid of Leptomeningeal Metastasis Patients: Functional Implication of miR-21 and Other Small RNAs in Cancer Malignancy

**Kyue-Yim Lee, Yoona Seo, Ji Hye Im, Jiho Rhim, Woosun Baek, Sewon Kim, Ji-Woong Kwon, Byong Chul Yoo, Sang Hoon Shin, Heon Yoo, Jong Bae Park, Ho-Shin Gwak and Jong Heon Kim**

## Supplementary Materials and Methods

### Nanoparticle Tracking Analysis (NTA) for Monitoring the Size and Number of EVs

After two sequential centrifugations, CSF samples were directly analyzed for the quantitative measurement of EV-sized particles using the NanoSight instrument (Model NTA NS300 with 642 nm red laser module, Malvern, Worcestershire, UK), which is a laser-based light scattering system that provides general nanoparticle characterization in terms of size and concentration ($10^6$ to $10^9$ particles/mL) as described previously [1]. Pure CSF samples were loaded into the laser module sample chamber manually with a confirmation of no air bubbles, and an automated camera module tracked the Brownian motion of particles in the liquid sample. The in-built software (NTA 3.2, Dev. Build 3.2.16, Malvern) calculated the EV size and concentration of triplicate of each sample with 30 s video capture. For the consistency of the observed values, we used the sCMOS (scientific Complementary metal-oxide-semiconductor) camera type and fixed camera level at 8 of 10, and set the temperature at 25°C.

### EV Protein Extraction and Western Blotting

EV protein of CSF samples was extracted using a Total Exosome RNA and Protein Isolation Kit according to the manufacturer's protocol (Thermo Fisher Scientific, Waltham, MA, USA). Briefly, pre-centrifuged CSF was directly mixed with an equal volume of extraction reagent and suspended at 4°C overnight. Captured EVs were pelleted by centrifugation (10,000 × *g* for 1 h) and resuspended with 10–60 μL ice-cold exosome resuspension buffer or 1× phosphate-buffered saline. Samples were incubated for 5–10 min at room temperature to allow the pellet to dissolve and were gently pipetted up and down to thoroughly resuspend the sample. The resuspended exosome sample for protein analysis was used immediately after protein concentration measurement using a BCA protein assay kit (Pierce Biotechnology, Rockford, IL, USA). Equal amount of protein lysates were homogenated with RIPA [50 mM Tris-HCl pH 7.5, 150 mM NaCl, 1% sodium deoxycholate, 0.1% SDS, 1% Triton X-100, 5 mM NaF, 2 mM sodium orthovanadate, 2 mM β-glycerophosphate, 2 mM EDTA, and protease inhibitor cocktail (Roche)], mixed with SDS gel-loading buffer, denatured for 5 min at 95°C and resolved by 4–20% SDS-PAGE. After electrophoresis, proteins on gel were transferred onto nitrocellulose membrane (Pall, Port Washington, NY, USA) or PVDF (Merck Millipore, Burlington, MA, USA), blocked with 5% nonfat dry milk in TBST (20 mM Tris-HCl pH 7.4, 150 mM NaCl, and 0.05% Tween 20) followed by overnight incubation at 4°C with primary antibodies against exosome membrane and cytosolic proteins. Membranes were washed and incubated with HRP-conjugated secondary antibodies. As a secondary antibody, horseradish peroxidase-conjugated anti-rabbit (Vector Laboratories, Burlingame, CA, USA) and anti-mouse IgG (Vector Laboratories) were used. Enhanced chemiluminescence signal were

detected and quantified by C-DIGIT (LI-COR, Lincoln, NE, USA) and/or X-ray film (AGFA, Mortsel, Belgium) exposure. Primary antibodies used through all western blotting were anti-GM-130 (mouse monoclonal, 610822, BD Biosciences, San Jose, CA, USA), anti-Flotillin-1 (rabbit polyclonal, ab41927, Abcam, Cambridge, UK), anti-CD63 (mouse monoclonal, MX-49.129.5, sc-5275, Santa Cruz Biotechnology, Santa Cruz, CA, USA), anti-CD81 (mouse monoclonal, B-11, sc-166029, Santa Cruz Biotechnology), anti-CD9 (rabbit polyclonal, 20597-1-AP, Proteintech, Rosemont, IL, USA), anti-cytochrome c (mouse monoclonal, 556433, BD Biosciences), anti-PTEN (rabbit monoclonal, 138G6, #9559, Cell Signaling Technology, Danvers, MA, USA), anti-PDCD4 (rabbit monoclonal, D29C6, #9535, Cell Signaling Technology), anti-Bcl-2 (mouse monoclonal, sc-509, Santa Cruz Biotechnology), anti-Spry2 (rabbit polyclonal, 07-524, Upstate; Sigma-Aldrich, St. Louis, MO, USA), and anti-Histone H3 (rabbit monoclonal, D1H2, #4499, Cell Signaling Technology).

**Cell Culture**

Human embryonic kidney cell line 293T, human glioma cell line U87MG, and A172 were obtained from the American Type Culture Collection (Manassas, VA, USA). Human chronic myelogenous leukemia K562 and human non-small cell lung cancer A549 cells were obtained from the Korean Type Culture Collection (Seoul, Korea). Lenti-X 293T cells were purchased from Takara Bio Inc. 293T, Lenti-X 293T, U87MG, and A172 cells were cultured in Dulbecco's modified Eagle medium (DMEM, HyClone; GE Healthcare, Chicago, IL, USA). A549 and K562 cells were cultured in RPMI-1640 media (HyClone). All media were supplemented with 10% fetal bovine serum (HyClone), 1% penicillin/streptomycin (Welgene, Gyeongsan, Korea) and 10 μg/mL ciprofloxacin (Santa Cruz Biotechnology).

**Identification of Known miRNA Reads and Statistical Analysis of Differential Expression**

Sequence alignment and detection of known and novel miRNAs were performed using miRDeep2 software algorithm. Prior to performing sequence alignment, the homo sapiens reference genome release hg19 was retrieved from UCSC genome browser and indexed using Bowtie (1.1.2), a bowtie for aligning sequencing reads to reference sequences. Those reads were then aligned homo sapiens mature and precursor miRNAs obtained from miRBase v21. The miRDeep2 algorithm is based on the miRNA biogenesis model; it aligns reads to potential hairpin structures in a manner consistent with Dicer processing and assigns scores that represent the probability that hairpins are true miRNA precursors. In addition to detecting known and novel miRNAs, miRDeep2 estimates their abundance. Raw data (the reads for each miRNA) were normalized by Relative Log Expression (RLE) normalization using DESeq2. For pre-processing, miRNAs with zeroed count across more than 50% of all samples are excluded leaving 108 mature miRNAs to be analyzed. We added 1 with normalized read count of the filtered miRNAs to facilitate log2 transformation to draw the correlation plot. For each miRNA, baseMean and log fold change were calculated between case and control. Statistical hypothesis test for comparison of two groups was conducted using the negative binomial Wald test in DESeq2. Differentially expressed miRNAs between two groups were determined by adjusting $|\text{fold change}| \geq 2$ & $p\text{-value} < 0.05$.

**Novel miRNAs Prediction**

Novel miRNAs were predicted from mature, star and loop sequence according to the RNAfold algorithm using miRDeep2. The RNAfold function uses the nearest-neighbor thermodynamic model to predict the minimum free-energy secondary structure of an RNA sequence. RNAfold-generated graphic contains the actual in sili-

co-folded hairpin, with the number of reads for each part of the hairpin, score for minimum free energy, score for randfold, and score for conserved seed sequence.

**Lentivirus Production, Infection and Establishment of Stable Cell Lines**

Lentivirus production was performed as previously reported. Briefly, ~$1.5 \times 10^6$ of 293FT, 293T, or Lenti-X 293T cells were plated on 100 mm culture dish 48 h before transfection. Then, 4.5 μg of lentiviral construct, 3 μg of psPAX2 (Addgene, Watertown, MA, USA), and 1.5 μg of pMD2.G (Addgene) were co-transfected into prepared cells using 27 μL of Lipofectamine 2000 (Thermo Fisher Scientific) or Metafectene PRO (Biontex, Munich, Germany) in OPTI-MEM (Thermo Fisher Scientific). The medium was changed with 5 ml culture media without antibiotics 5 h after transfection. After 24 h, caffeine (final concentration, 2 mM) was supplemented in each plate, and then the medium containing lentivirus was harvested 48 h after transfection. Viral particles containing media were filtered through 0.45 μm Acrodisc Syringe Filters with Supor Membrane (Pall) and concentrated using Lenti-X concentrator (Takara Bio Inc.). For the lentivirus infection, $2 \times 10^5$ of cells were plated on 35 mm culture dishes 48 h before infection and half of purified lentivirus were added onto the target cells in the presence of 10 μg/mL polybrene (Sigma-Aldrich). After incubating for 12–24 h, target cells were recovered 9–24 h with changing complete medium, lentivirus transduced cells were selected with blasticidin S (5 μg/mL; Invivogen, San Diego, CA, USA) for 2–3 days.

**Transient Transfection of RNA Oligomers and Migration Assay**

Synthetic control and miRNA mimic for miR-21 were purchased from Bioneer (Daejeon, Korea). $6 \times 10^5$ of A549 cells were plated on 6-well cell culture plates 48 h before scratching and transfection. Then 4–6 lines were scratched in each confluent monolayer with a sterile 200 μl tip. Dislodged cells were removed by washing with warm Dulbecco's phosphate-buffered saline, and representative lines with similar width were selected and photographed in each group at starting point. After then, 100 picomole of each RNA oligomer and 5 μL of Lipofectamine 2000 were used for each transient transfection. After 48 h, snapshots of the scratch were taken with the microscope (100×) (Axio Observer, Zeiss, Oberkochen, Germany).

**Small RNA Analysis by Splinted Ligation**

Total RNAs were isolated with TRIzol (Invitrogen; Thermo Fisher Scientific) and splinted ligation was performed as described previously [2,3]. In brief, 10 fmol of control synthetic miR-21-5p RNA, 200 ng of total RNA from LM CSF EVs, and K562 cells were incubated in capture mixture (total 9 μL volume; 1.5 μL 10× capture buffer, 0.1 pmol [$^{32}$P] 5′-end-labeled ligation oligomer, 0.1 pmol bridge oligonucleotide). Capture reaction was performed in PCR machine and the reaction parameters were as follows: 95°C for 1 min, 65°C for 2 min, and 37°C for 10 min. After the capture reaction 1 μL of T4 DNA ligase (Affymetrix-USB; Thermo Fisher Scientific) and 10 μL of 2× ligase buffer were added in the capture reaction mixture. The ligation reaction was performed in the PCR machine again and the reaction parameters were as follows: 30°C for 1 h, and 75°C for 10 min. Reaction was terminated by adding 1 μL of shrimp alkaline phosphatase (Affymetrix-USB; Thermo Fisher Scientific; 1 U/μL) and further incubated at 37°C for 10 min. Reaction mixtures were resolved on denaturing urea-12% PAGE and radioactive signals were scanned by the BAS-2500 analyzer (Fujifilm, Tokyo, Japan) or obtained from X-ray film (AGFA) exposure at -80°C. hsa-miR-21-5p RNA and ligation oligonucleotide were synthesize from Integrated DNA Technologies (Coralville, IA, USA); hsa-miR-21-5p RNA, 5′-UAGCUUAUCAGACUGAUGUUGA-3′; ligation oligonucleotide 5′-CGCTTATGACAT TC/dideoxyC/-3′; hsa-miR-21-5p bridge oligonucleotide (Macrogen, Seoul, Korea); 5′-GAATGTCATAAGCGTCAACATCAGTCTGATAAGCTA-3′.

**Table S1.** Experimental raw data of the CSF samples in this study. See Excel document.

**Table S2.** Clinical characteristics of additional CSF samples and their applications in this study (*n* = 11).

| Patients No. | Gender | Age | Patient group | Primary disease | Sample site | Applications |
|---|---|---|---|---|---|---|
| LM15 | Male | 71 | LM | NSCLC | Lumbar | ddPCR (E.V.) |
| LM16 | Male | 69 | LM | NSCLC | Lumbar | ddPCR (E.V.) |
| LM17 | Female | 49 | LM | Breast cancer | Lumbar | ddPCR (E.V.) |
| LM18 | Female | 54 | LM | NSCLC | Intraventricular | ddPCR (E.V.) |
| LM19 | Female | 49 | LM | Breast cancer | Intraventricular | ddPCR (E.V.) |
| HC11 | Female | 72 | Healthy Control | Unruptured an | Intraventricular | ddPCR (E.V.) |
| HC12 | Female | 63 | Healthy Control | Unruptured an | Cisternal | ddPCR (E.V.) |
| HC13 | Female | 68 | Healthy Control | Unruptured an | Cisternal | ddPCR (E.V.) |
| HC14 | Female | 45 | Healthy Control | Unruptured an | Lumbar | ddPCR (E.V.) |
| HC15 | Male | 69 | Healthy Control | Unruptured an | Intraventricular | ddPCR (E.V.) |
| HC16 | Male | 59 | Healthy Control | Unruptured an | Lumbar | ddPCR (E.V.) |

LM, leptomeningeal metastasis; HC, healthy control; NSCLC, non-small cell lung cancer; Unruptured an, unruptured aneurysms; ddPCR, droplet digital PCR; E.V., external validation.

**Table S3.** Oligonucleotides used in ddPCR analysis. Fwd, forward oligomer; Rev, reverse oligomer.

| Target | Sequence of oligonucleotides | |
|---|---|---|
| miR-423-5p | Fwd: | 5′-TGA GGG GCA GAG AGC GAG ACT T-3′ |
| | Rev: | 5′-CCA CTA CGC CTC CGC TTT CCT CTC TAT GG-3′ |
| miR-1273g-3p | Fwd: | 5′-ACC ACT GCA CTC CAG CCT GAG-3′ |
| | Rev: | 5′-CCA CTA CGC CTC CGC TTT CCT CTC-3′ |
| miRNA-4271 | Fwd: | 5′-GGG GGA AGA AAA GGT GGG-3′ |
| | Rev: | 5′-CCA CTA CGC CTC CGC TTT CCT CTC TAT GG-3′ |
| piRNA-33415 | Fwd: | 5′-GTT CGA TCC CCG TAC GGG CCA CC-3′ |
| | Rev: | 5′-CCA CTA CGC CTC CGC TTT CCT CTC TAT GG-3′ |
| piRNA-36340 | Fwd: | 5′-TCG TTT CCG GCT CGA AGG ACC-3′ |
| | Rev: | 5′-CCA CTA CGC CTC CGC TTT CCT CTC TAT GG-3′ |
| Y RNA-Y69 | Fwd: | 5′-CTT CTC ACT ACT GCA CTT GAC-3′ |
| | Rev: | 5′-CCA CTA CGC CTC CGC TTT CCT CTC TAT GG-3′ |
| snRNA_200C70 | Fwd: | 5′-GCA TTG GCA ATT TTT GAC AG-3′ |
| | Rev: | 5′-CCA CTA CGC CTC CGC TTT CCT CTC -3′ |
| vtRNA_6C57F3 | Fwd: | 5′-TCT GGG TGG TTC GAG ACC-3′ |
| | Rev: | 5′-CCA CTA CGC CTC CGC TTT CCT CTC-3′ |
| novel_miRNA-973 | Fwd: | 5′-GTT CGA ATC CTT CCT CCC-3′ |
| | Rev: | 5′-CCA CTA CGC CTC CGC TTT CCT CTC-3′ |
| scRNA_F3729 | Fwd: | 5′-AGC TCA GCG GTT ACT TCG AC-3′ |
| | Rev: | 5′-CCA CTA CGC CTC CGC TTT CCT CTC TAT GG-3′ |

**Table S4.** List of smRNA sequencing expression raw data by descending fold change and *p*-value. See Excel document.

## Supplementary Figures

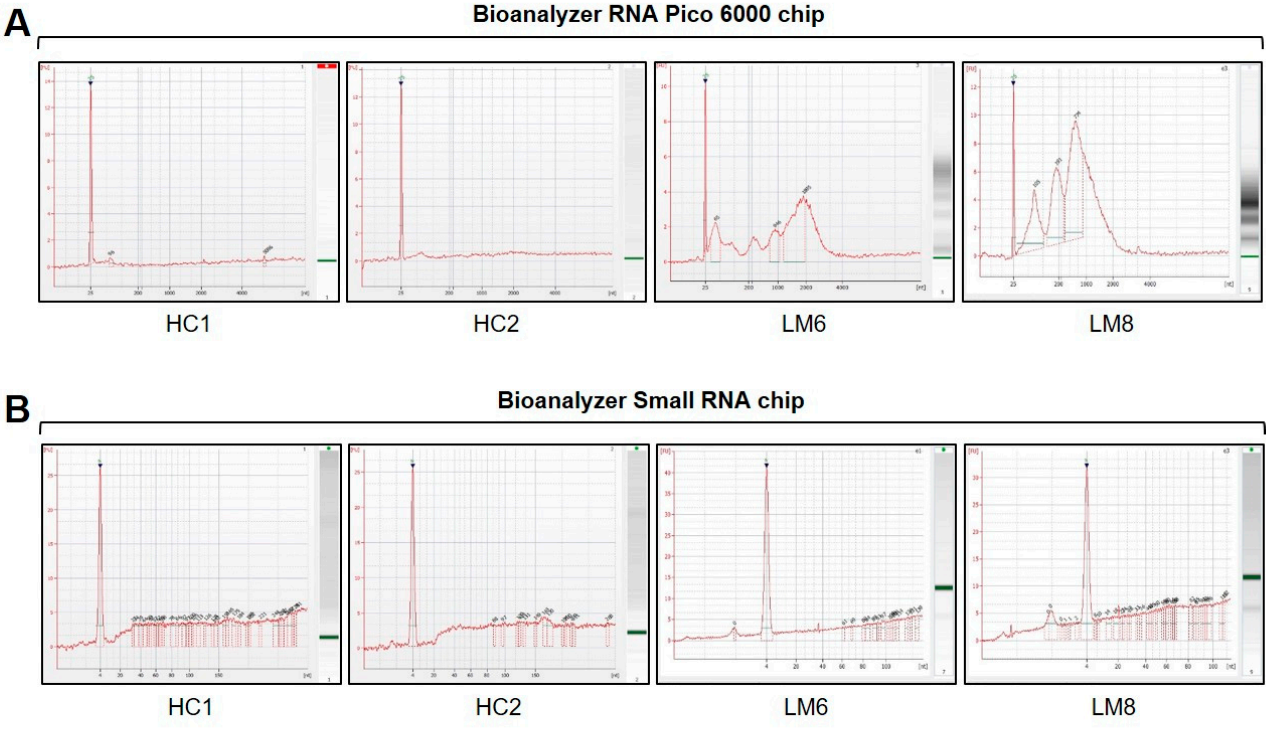


**Figure S1.** Bioanalyzer analysis of the size distribution of RNA from HC and LM CSF EVs. The Pico 6000 chip analysis for total EV RNA (< 6,000 nucleotides, **A**) and Small RNA chip analysis for smRNA of EV (< 200 nucleotides, **B**) by Agilent 2100 Bioanalyzer.

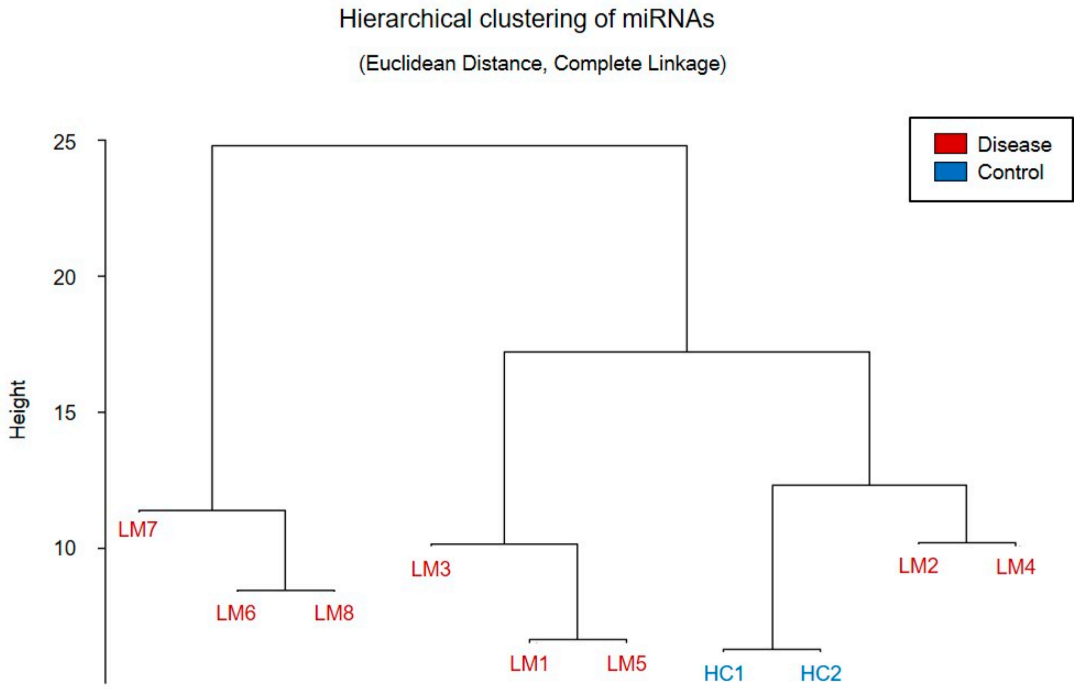


**Figure S2.** Hierarchical clustering dendrogram of miRNAs among LM patients. Analysis of relative miRNA expression profile in EVs extracted from LM and HC. Hierarchical clustering dendrogram indicates the Euclidean distance indicating similarity of significantly expressed miRNA.

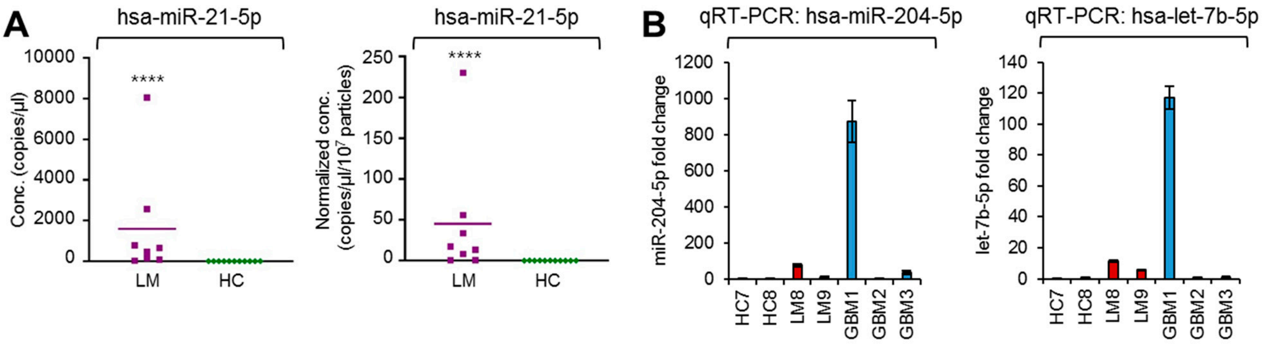


**Figure S3.** Biochemical verification of biased miRNA expression in EVs derived from LM and HC by ddPCR and qRT-PCR. (**A**) Analysis of upregulated miR-21-5p levels by ddPCR in EVs from additional LM (purple rectangle, $n = 8$) and HC (green rhombus, $n = 11$) (left graph). ddPCR concentration was also normalized by the initial EV particle numbers of each samples obtained from NTA, and shown as copies/μl per $10^7$ particles (right graph). (The Mann-Whitney U test, **** $p < 0.0001$). (**B**) Analysis of biased expression level of miRNAs (TaqMan Advanced miRNA Assay probes; hsa-miR-204-5p and hsa-let-7b-5p) by qRT-PCR in CSF EVs from 2 LM (LM8 and LM9) and 2 HC (HC7 and HC8), and 3 glioblastoma multiforme patients (GBM1-3). The data represent the mean values of three independent experiments ($n = 3$) and error bars in the graph represent ± standard deviation.

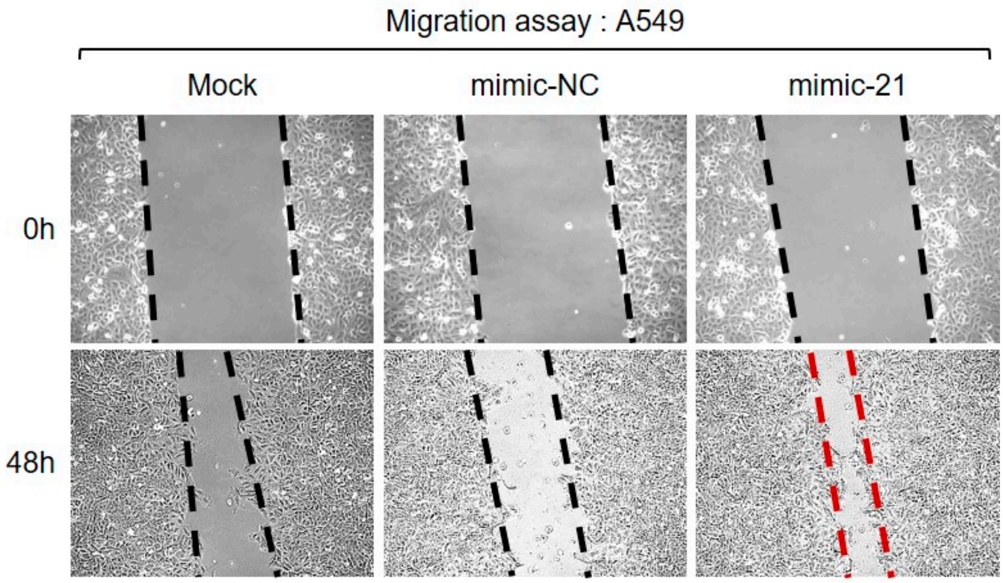


**Figure S4.** Investigation of synthetic miR-21 functionality on cellular migratory phenotype of NSCLC A549 cells. Representative images of migration assays after 48 h transfection with control (mimic-NC) or synthetic miR-21 (mimic-21) mimics to A549 cells. Phase contrast microscopy images were taken with Axio Observer (100×, Zeiss).

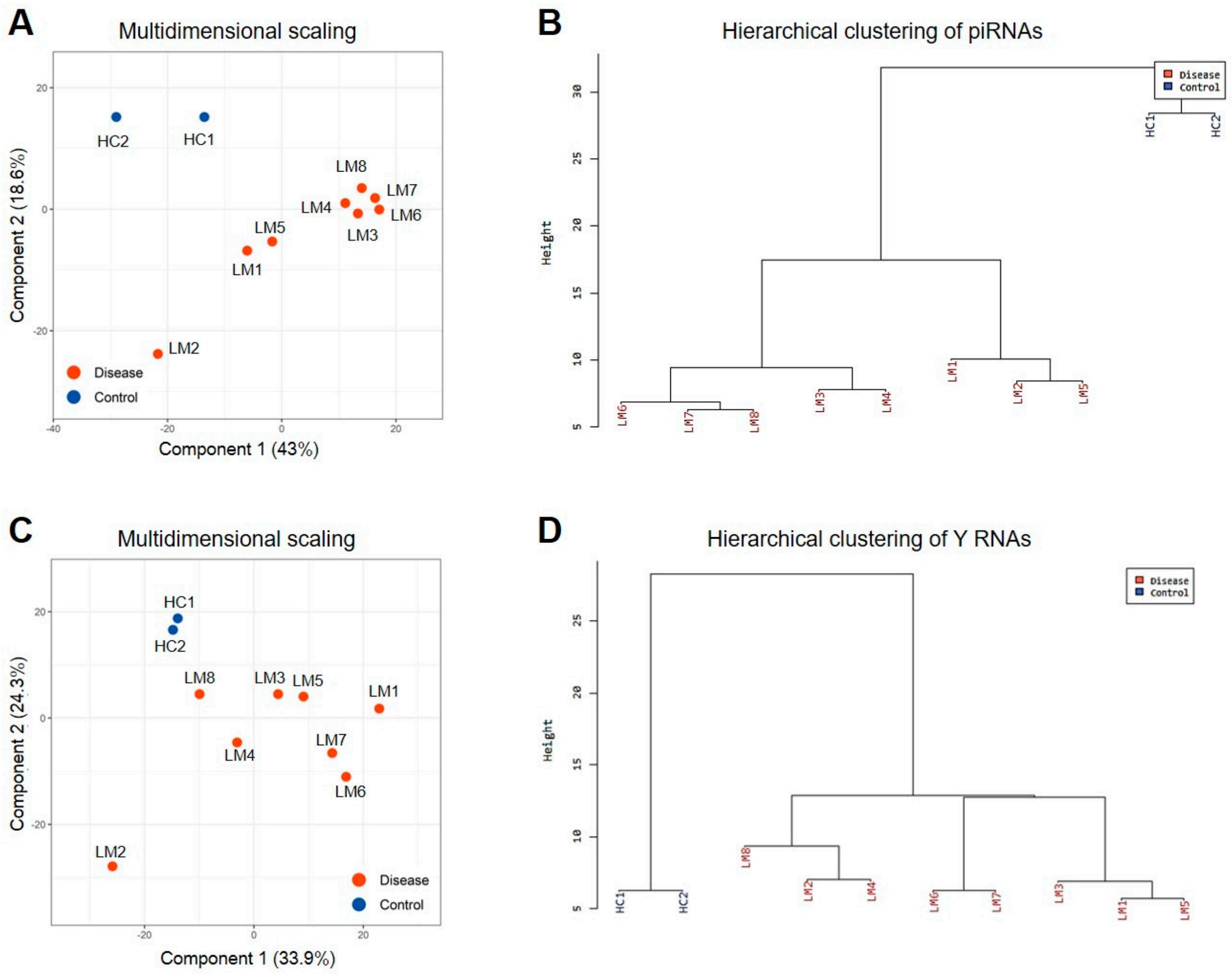


**Figure S5.** Hierarchical clustering analysis of piRNAs and Y RNAs in EVs from LM patient CSF**.** Analysis of relative piRNA and Y RNA expression profile in EVs extracted from LM and HC. Hierarchical clustering analysis of significantly expressed piRNA was visualized via (**A**) Multidimensional scaling (MDS) map of HC and LM was generated with proximity calculated in Euclidean distance and (**B**) clustering dendrogram distances indicating similarity. Hierarchical clustering analysis of significantly expressed Y RNA was also visualized via (**C**) MDS map of HC and LM was generated with proximity calculated in Euclidean distance and (**D**) clustering dendrogram distances indicating similarity.

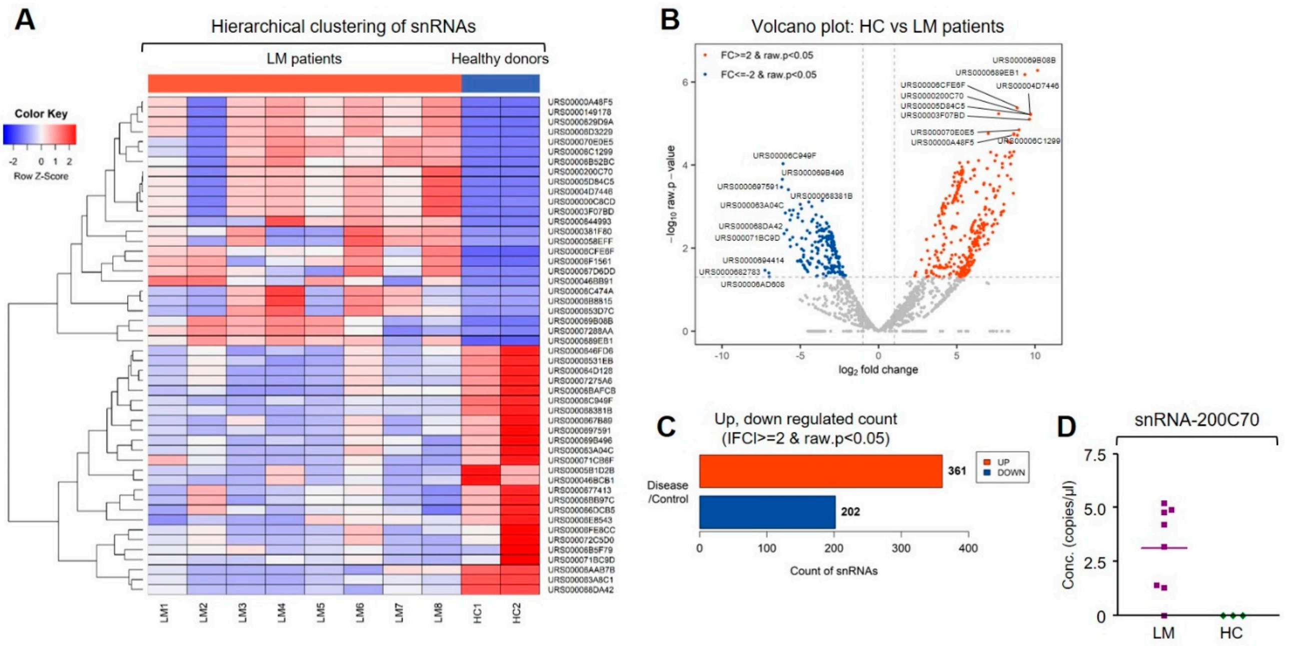


**Figure S6.** Distinct expression of snRNAs in EVs from LM patient CSF**.** Analysis of relative snRNAs expression profile in EVs extracted from LM and HC. Hierarchical clustering analysis of significantly expressed snRNA was visualized via (**A**) heatmap showing z score of extravesicular snRNA from HC ($n$ = 2) and patients with LM ($n$ = 8) with 25 upregulated and 25 downregulated snRNA that best satisfied FC2 value and adjusted $p$-value. (**B**) Volcano plot shows differentially expressed snRNA in HC and LM patients with the x-axis showing log2 fold-change and y-axis showing -log10 of the raw $p$-value from LM versus HC snRNA expression counts. (**C**) Count of up- and downregulated snRNA was found by fold change and raw $p$-value. (**D**) An upregulated snRNA was confirmed in HC ($n$ = 3) and LM EVs ($n$ = 8) using ddPCR (Mann-Whitney U test).

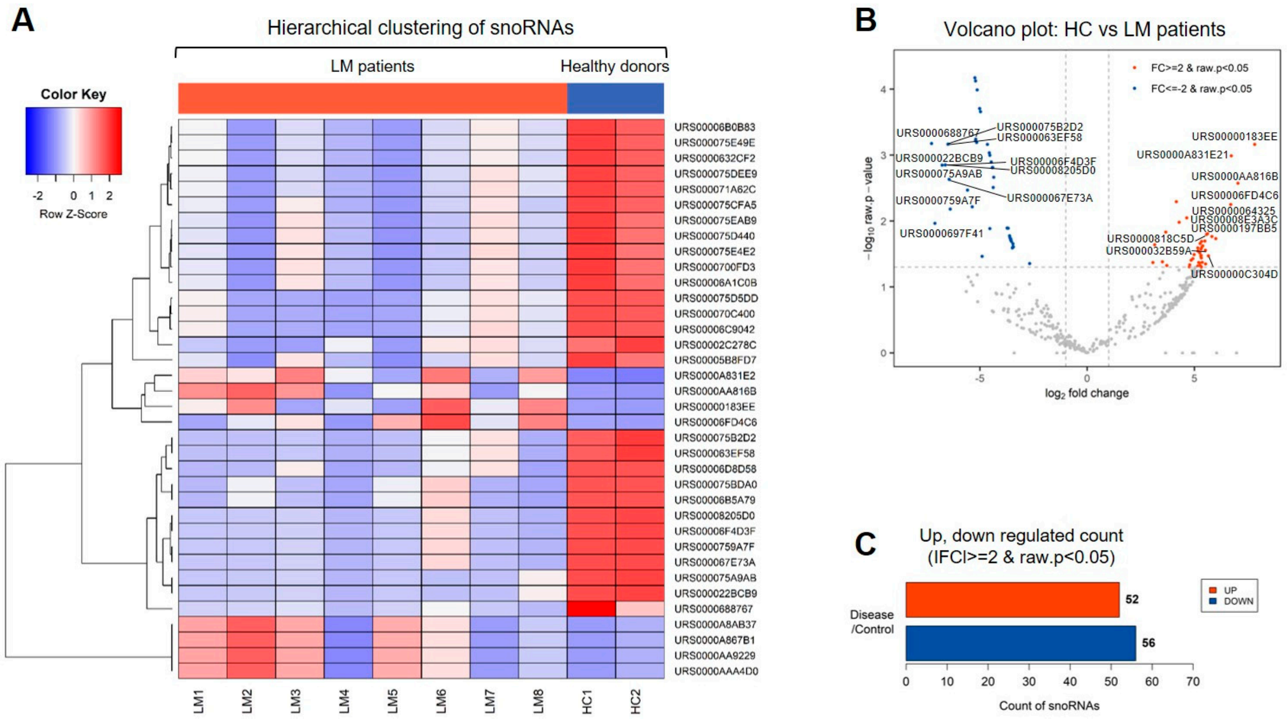


**Figure S7.** Distinct expression of snoRNAs in EVs from LM patient CSF**.** Analysis of relative snoRNAs expression profile in EVs extracted from LM and HC. Hierarchical clustering analysis of significantly expressed snoRNA was visualized via (**A**) heatmap showing z score of extravesicular snoRNA from HC ($n$ = 2) and patients with LM ($n$ = 8) with 36 snoRNA satisfying FC2 value and adjusted $p$-value. (**B**) Volcano plot shows differentially expressed snoRNA in HC and LM patients with the x-axis showing log2 fold-change and y-axis showing -log10 of the raw $p$-value from LM versus HC snoRNA expression counts. (**C**) Count of up- and downregulated snoRNA was found by fold change and raw $p$-value.

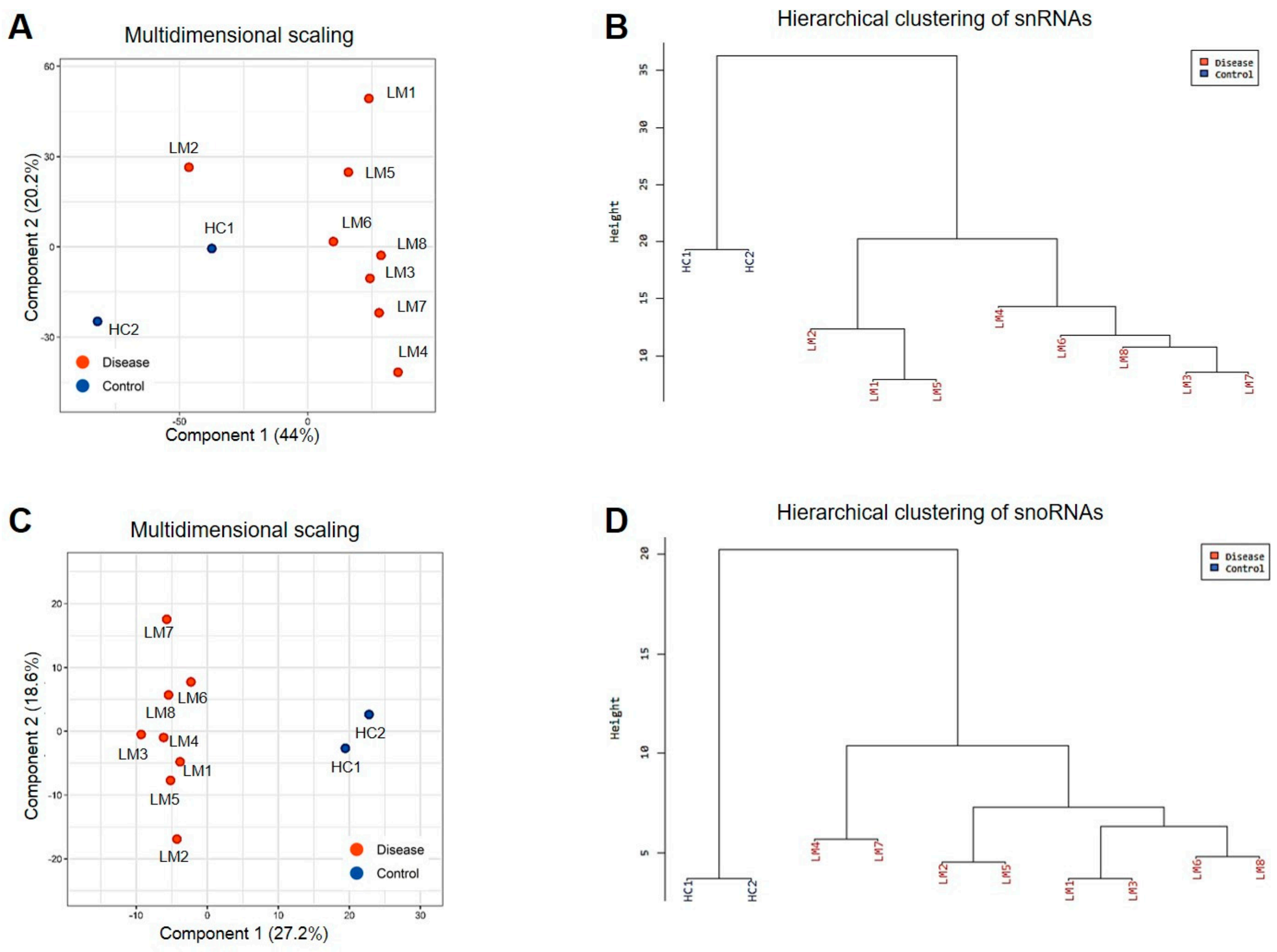


**Figure S8.** Hierarchical clustering analysis of snRNAs and snoRNAs in EVs from LM patient CSF. Analysis of relative snRNA and snoRNA expression profile in EVs extracted from LM and HC. Hierarchical clustering analysis of snRNA was visualized via (**A**) MDS map of HC and LM generated with proximity calculated in Euclidean distance and (**B**) clustering dendrogram distances indicating similarity. Hierarchical clustering analysis of snoRNA was also visualized via (**C**) MDS map of HC and LM generated with proximity calculated in Euclidean distance and (**D**) clustering dendrogram distances indicating similarity.

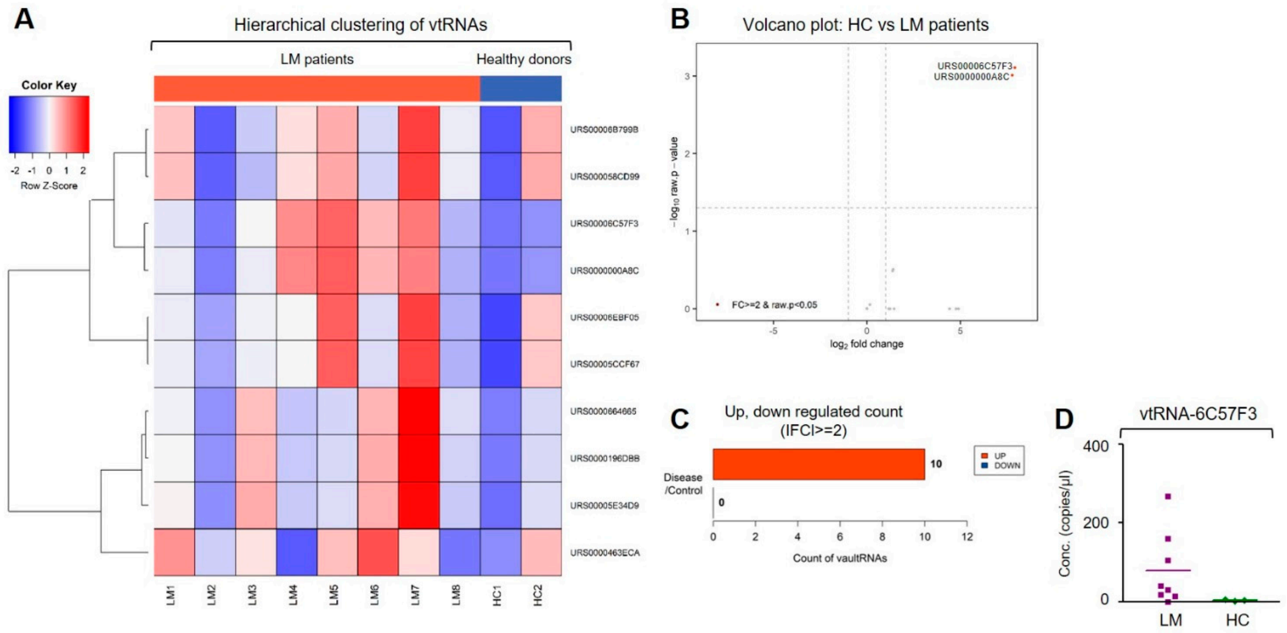


**Figure S9.** Distinct expression of vtRNAs in EVs from LM patient CSF**.** Analysis of relative vtRNAs expression profile in EVs extracted from LM and HC. Hierarchical clustering analysis of vtRNA was visualized via (**A**) heatmap showing z score of extravesicular vtRNA from HC ($n$ = 2) and patients with LM ($n$ = 8) of 10 vtRNA satisfying FC2 value. (**B**) Volcano plot shows differentially expressed vtRNA in HC and LM patients with the x-axis showing log2 fold-change and y-axis showing -log10 of the raw $p$-value from LM versus HC vtRNA expression counts. (**C**) Count of upregulated vtRNA was found by fold change value. (**D**) An upregulated vtRNA-6C57F3 was confirmed in HC ($n$ = 3) and LM EVs ($n$ = 8) using ddPCR (Unpaired $t$-test with Welch's correction).

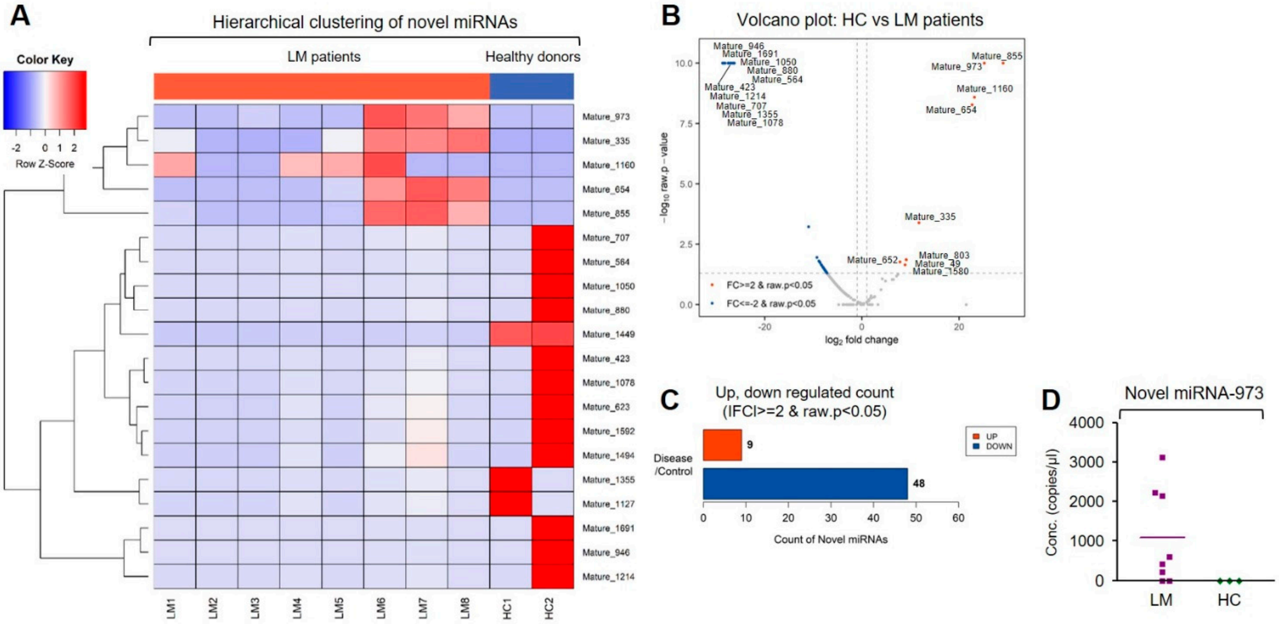


**Figure S10.** Distinct expression of novel miRNAs in EVs from LM patient CSF**.** Analysis of relative novel miRNAs expression profile in EVs extracted from LM and HC. Hierarchical clustering analysis of novel miRNAs was visualized via (**A**) heatmap showing z score of extravesicular novel miRNA from HC ($n$ = 2) and patients with LM ($n$ = 8) of 20 novel miRNA satisfying FC2 value and adjusted $p$-value. (**B**) Volcano plot shows differentially expressed novel miRNA in HC and LM patients with the x-axis showing log2 fold-change and y-axis showing -log10 of the raw $p$-value from LM versus HC novel miRNA expression counts. (**C**) Count of up- and downregulated novel miRNA was found by fold change and raw $p$-value. (**D**) An upregulated novel miRNA-973 was confirmed in HC ($n$ = 3) and LM EVs ($n$ = 8) using ddPCR (Unpaired $t$-test with Welch's correction).

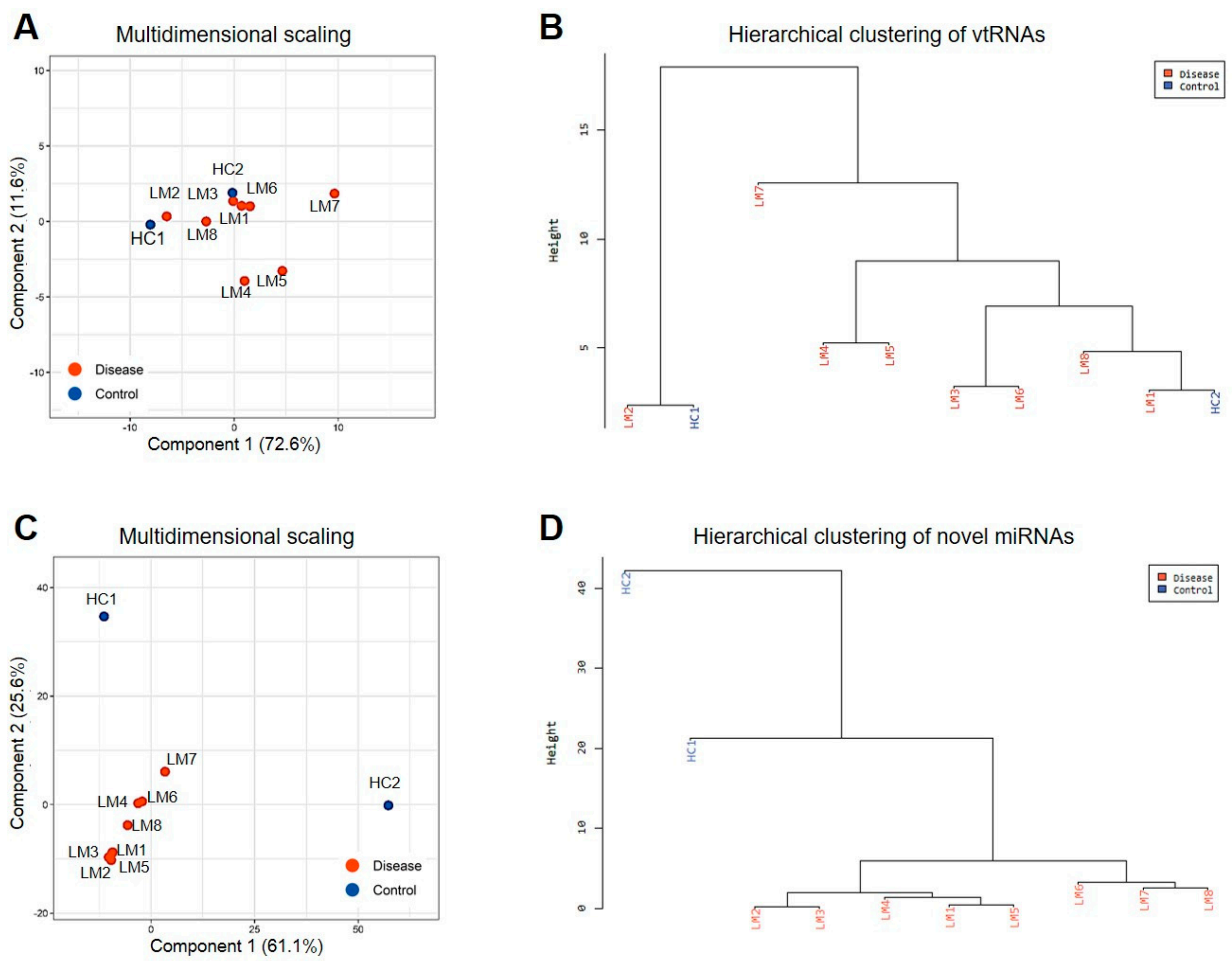


**Figure S11.** Hierarchical clustering analysis of vtRNAs and novel miRNAs in EVs from LM patient CSF**.** Analysis of relative vtRNA and novel miRNA expression profile in EVs extracted from LM and HC. Hierarchical clustering analysis of vtRNA was visualized via (**A**) MDS map of HC and LM was generated with proximity calculated in Euclidean distance and (**B**) clustering dendrogram distances indicating similarity. Hierarchical clustering analysis of novel miRNA was also visualized via (**C**) MDS map of HC and LM was generated with proximity calculated in Euclidean distance and (**D**) clustering dendrogram distances indicating similarity.

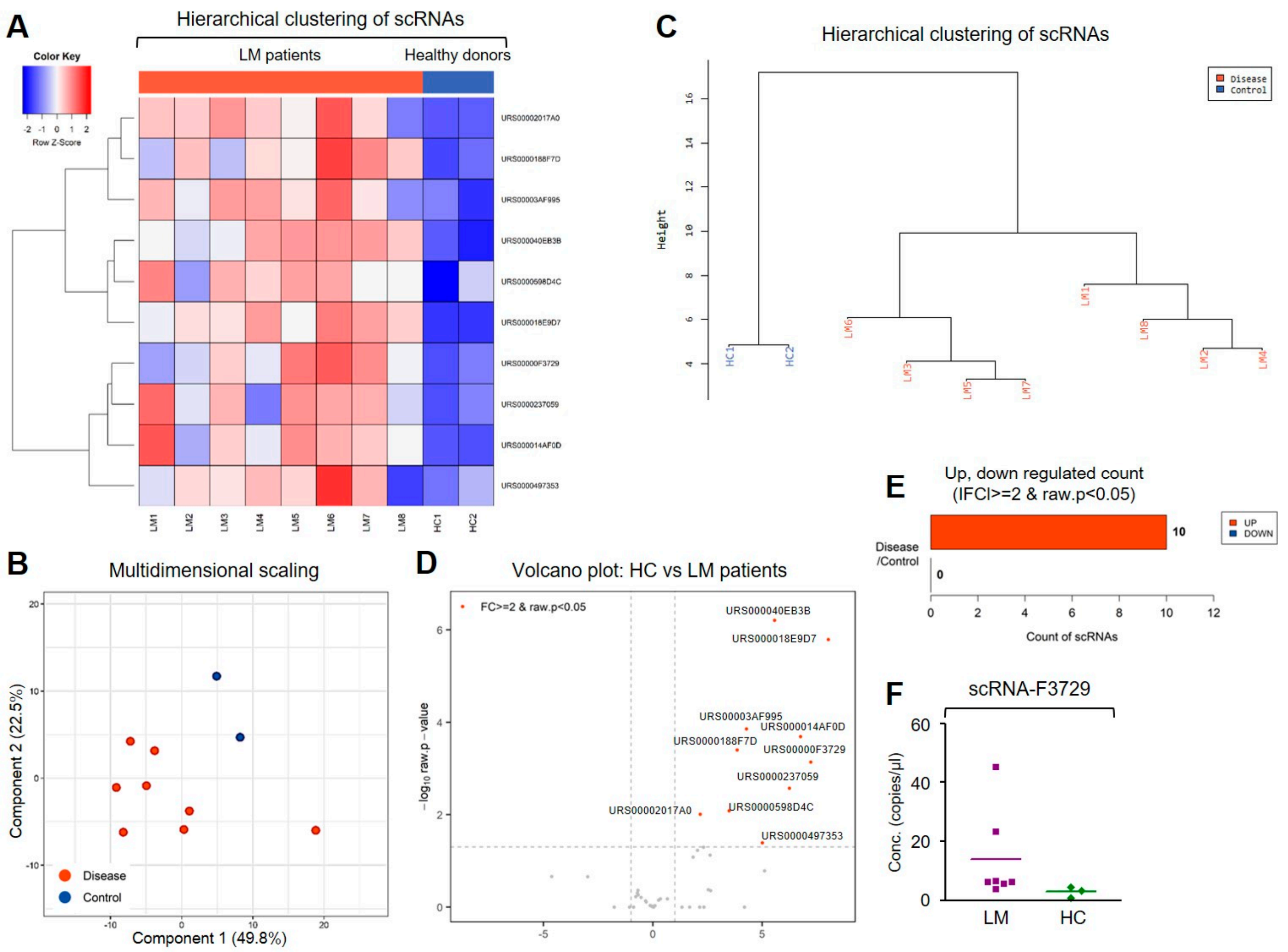


**Figure S12.** Distinct expression of scRNAs in EVs from LM patient CSF**.** Analysis of relative scRNAs expression profile in EVs extracted from LM and HC. Hierarchical clustering analysis of scRNAs was visualized via (**A**) heatmap showing z score of extravesicular scRNA from HC (*n* = 2) and patients with LM (*n* = 8) of 10 scRNA satisfying FC2 value and raw *p*-value. (**B**) MDS map of HC and LM was generated with proximity calculated in Euclidean distance and (**C**) clustering dendrogram distances indicating similarity. (**D**)Volcano plot shows differentially expressed scRNA in HC and LM patients with the x-axis showing log2 fold-change and y-axis showing -log10 of the raw *p*-value from LM versus HC scRNA expression counts. (**E**) Count of upregulated scRNA was found by fold change and raw *p*-value. (F) An upregulated scRNA-F3729 was confirmed in HC (*n* = 3) and LM EVs (*n* = 8) using ddPCR (Unpaired *t*-test with Welch's correction).

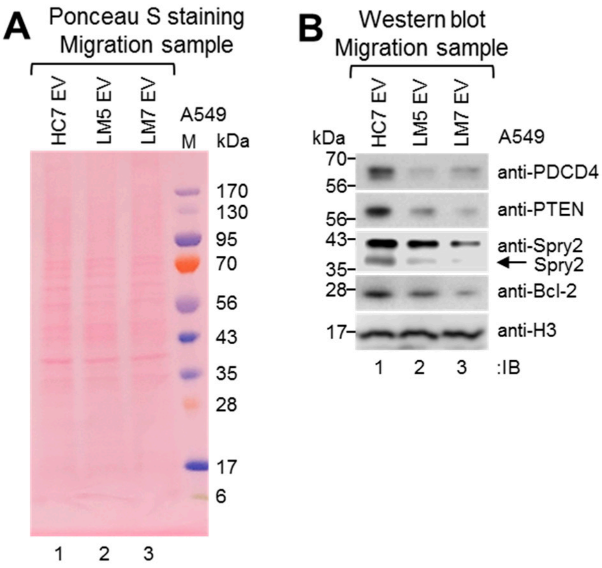


**Figure S13.** Western blot analysis of the validated target genes of miR-21 in LM CSF EV-treated A549 migration assay samples. (**A**) Ponceau S-stained total proteome of migration assay samples in Figure 6D. (**B**) Western blot analysis of validated miR-21 target genes in migration assay samples. The expression of PDCD4, PTEN, Spry2, and Bcl-2 protein was markedly downregulated in LM patient CSF EV-treated A549 groups compared with HC.

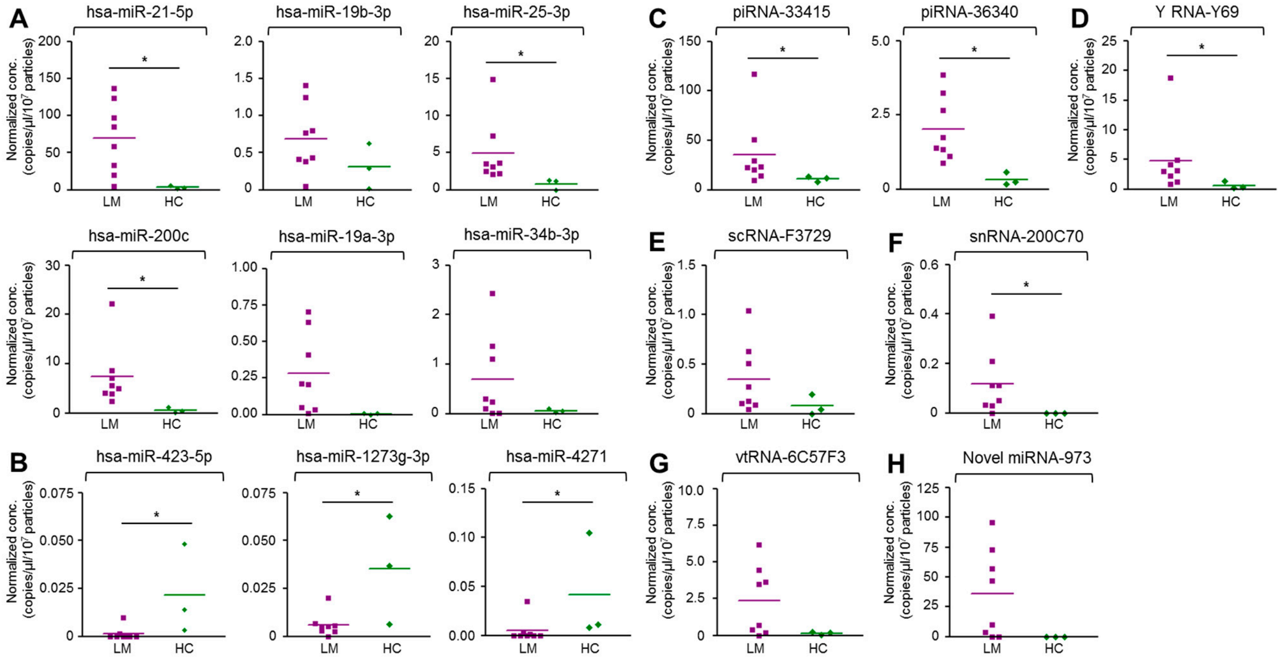


**Figure S14.** Biochemical verification of normalized smRNA expression in EVs derived from LM and HC by ddPCR. Concentration of ddPCR was normalized by the initial EV particle numbers of each sample obtained from NTA and shown as copies/µl per $10^7$ particles. Normalized concentration of (**A**) 6 upregulated miRNAs, (**B**) 3 downregulated miRNAs, (**C**) two piRNAs, (**D**) a Y RNA, (**E**) a scRNA, (**F**) a snRNA, (**G**) a vtRNA, and (**H**) a novel miRNA were confirmed by ddPCR (Mann-Whitney U test, * $p < 0.05$). LM (purple rectangle, $n = 8$); HC (green rhombus, $n = 3$).

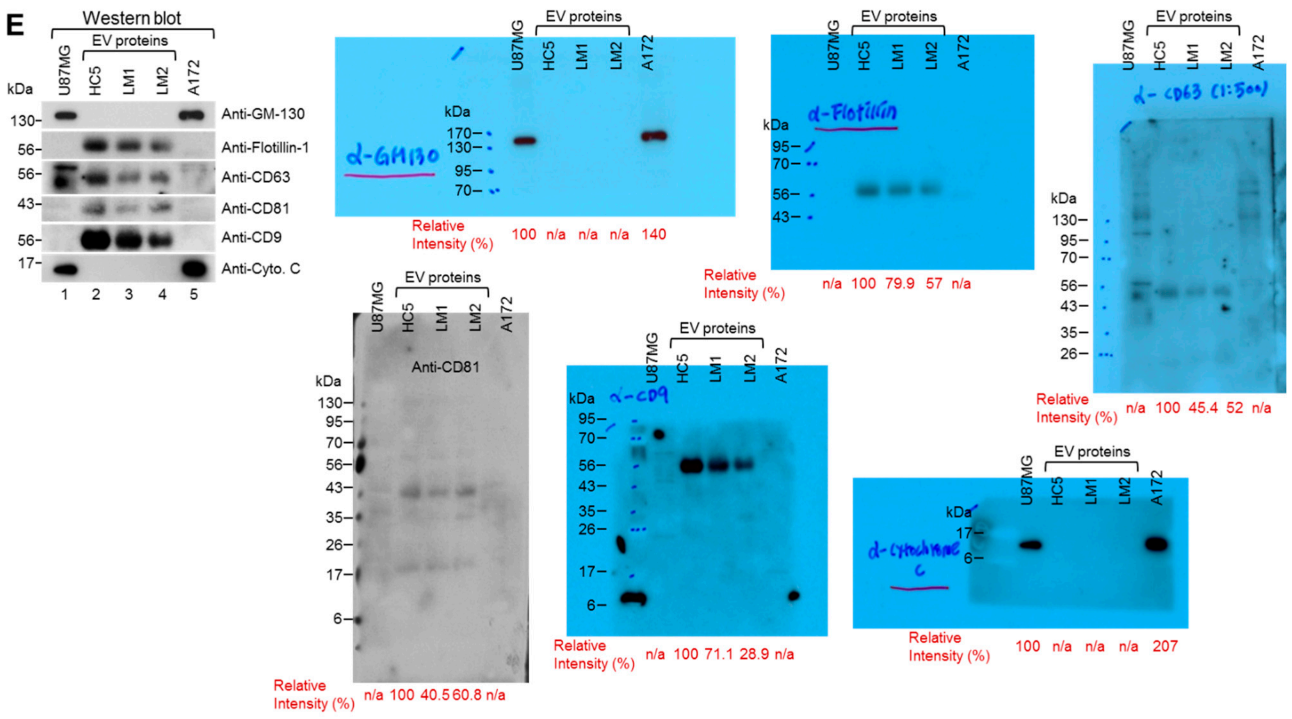


**Figure S15.** Original images of western blot related with Figure 1E.

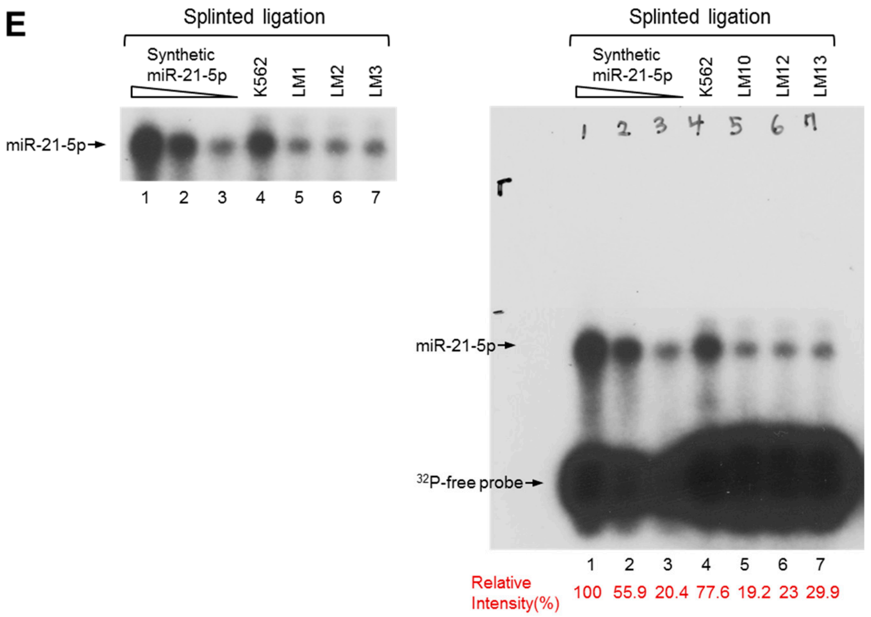


**Figure S16.** Original image of splinted ligation related with Figure 4E.

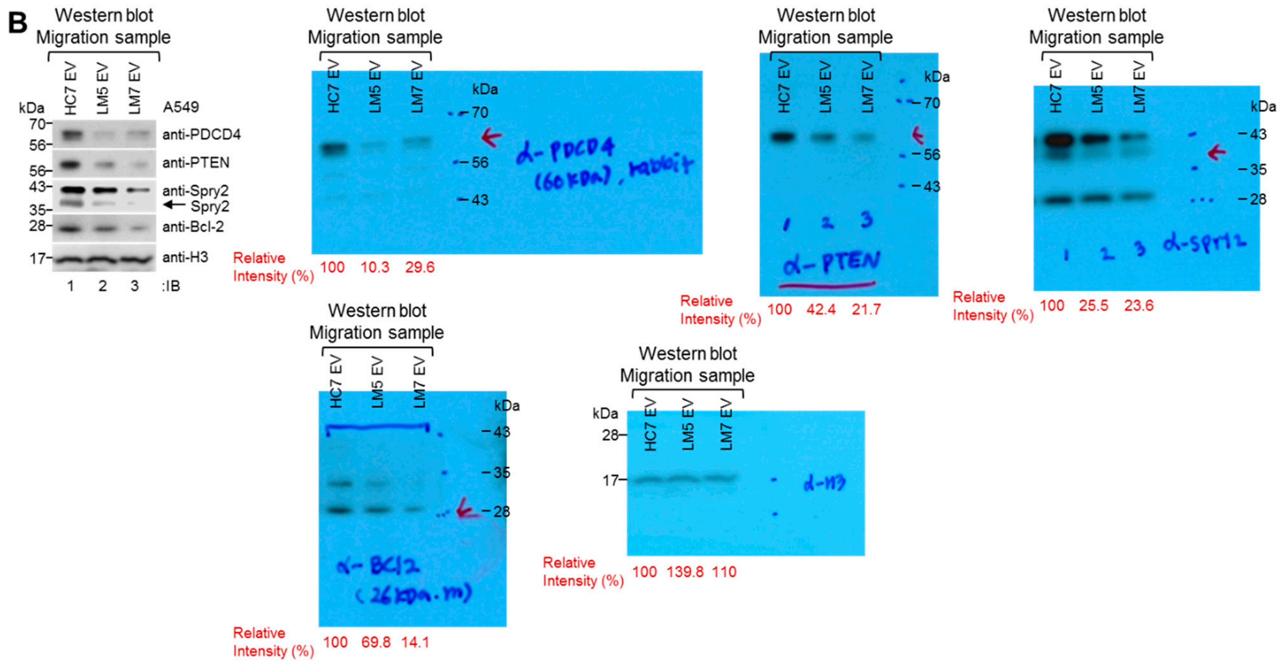


**Figure S17.** Original images of western blot related with Figure S13B.

## Supplementary References

1. Lee, K. Y.; Im, J. H.; Lin, W.; Gwak, H. S.; Kim, J. H.; Yoo, B. C.; Kim, T. H.; Park, J. B.; Park, H. J.; Kim, H. J.; et al. Nanoparticles in 472 Human Cerebrospinal Fluid: Changes in Extracellular Vesicle Concentration and miR-21 Expression as a Biomarker for Leptomeningeal Metastasis. *Cancers (Basel)* **2020,** 12, doi:10.3390/cancers12102745.
2. Yin, J.; Kim, T. H.; Park, N.; Shin, D.; Choi, H. I.; Cho, S.; Park, J. B.; Kim, J. H., TRIM71 suppresses tumorigenesis via modulation of Lin28B-let-7-HMGA2 signaling. *Oncotarget* **2016,** 7, 79854-79868, doi: 10.18632/oncotarget.13036.
3. Lee, S. H.; Cho, S.; Kim, M. S.; Choi, K.; Cho, J. Y.; Gwak, H. S.; Kim, Y. J.; Yoo, H.; Lee, S. H.; Park, J. B.; et al. The ubiquitin ligase human TRIM71 regulates let-7 microRNA biogenesis via modulation of Lin28B protein. *Biochim. Biophys. Acta* **2014,** 1839, 374–386, doi: 10.1016/j.bbagrm.2014.02.017.
